# Qualitative, semi-quantitative, and quantitative simulation of the osmoregulation system in yeast

**DOI:** 10.1016/j.biosystems.2015.04.003

**Published:** 2015-05

**Authors:** Wei Pang, George M. Coghill

**Affiliations:** School of Natural and Computing Sciences, University of Aberdeen, Aberdeen AB24 3UE, UK

**Keywords:** Osmotic stress response, Semi-quantitative simulation, Qualitative simulation

## Abstract

In this paper we demonstrate how *Morven*, a computational framework which can perform qualitative, semi-quantitative, and quantitative simulation of dynamical systems using the same model formalism, is applied to study the osmotic stress response pathway in yeast. First the *Morven* framework itself is briefly introduced in terms of the model formalism employed and output format. We then built a qualitative model for the biophysical process of the osmoregulation in yeast, and a global qualitative-level picture was obtained through qualitative simulation of this model. Furthermore, we constructed a *Morven* model based on existing quantitative model of the osmoregulation system. This model was then simulated qualitatively, semi-quantitatively, and quantitatively. The obtained simulation results are presented with an analysis. Finally the future development of the *Morven* framework for modelling the dynamic biological systems is discussed.

## 1 Introduction

Quantitative modelling approaches have been widely used in the field of systems biology. They offer precise descriptions of the dynamics of biological systems, provide deeper understanding of the underlying mechanisms, and can predict possible behaviours of the system. In particular, Ordinary Differential Equation (ODE) models are frequently used in modelling such complex biological systems. For example, the Klipp–Hohmann model (Klipp et al., 2005) of osmoregulation in *Saccharomyces cerevisiae*, a well-established model composed of 35 ODEs and 70 parameters, comprehensively describes the dynamics of the osmotic stress response pathway in *S. cerevisiae*.

However, there exist several challenges regarding modelling complex systems in systems biology: (1) it is often the case that only incomplete knowledge is known about the system, for instance, the relations between components of the system are not well understood. It is also possible in biology that some of the key components of the system are not even identified. (2) Even when the structure of the biological model is determined, it is very common that the values of some model parameters cannot be inferred confidently due to sparse and noisy experimental data. (3) Because of the nature of the biological systems or technical limitations initial conditions of some variables cannot be measured very precisely. (4) Given an existing quantitative model, qualitative behaviours of a system cannot be straightforwardly analysed, especially when the model structure is complex.

Quantitative modelling approaches tend to deal with the first issue by making additional assumptions on which components should be included in the model and relations between all model components. However, such assumptions are not easy, or even impossible, to verify, and this makes the resulting quantitative models less convincing and robust. For the second issue, many parameter estimation techniques have been developed, for instance, the recent work of Lillacci and Khammash (2010) and Tashkova et al. (2011). But these techniques cannot deal very well with situations where data are both sparse and noisy. As for the third issue, quantitative modellers tend to either assume precise measurements or estimate the initial conditions by statistical methods. Such assumptions and estimates may result in inaccurate predictions of the system, especially when the measurements are sparse and noisy. For the final issue, modellers may just focus on some local features and dynamics of the model, and often ignore the global picture underlying the dynamic biological system at higher levels of abstraction.

From the above observations, it is evident that there are limitations to using quantitative modelling approaches alone to deal with major challenges within the field of systems biology. This necessitates novel modelling and simulation approaches, either as independent tools or complementary methods, to better address these challenges.

In this paper, we present a solution to address the above-mentioned issues, the *Morven* framework (Coghill, 1996; Bruce and Coghill, 2005). This solution covers a broad spectrum of levels of abstraction: quantitative, semi-quantitative, and qualitative. The use of *Morven* facilitates the capture and analysis of system behaviours at varying levels, and can better deal with imperfect data and insufficient knowledge. More importantly, *Morven* is an integrated simulation package: a model is only built once and then can be used to perform all three kinds of simulations. This can reduce the amount of model-building work and help modellers better understand the system dynamics.

To test the validity of the *Morven* framework, we utilise *Morven* to model and simulate the osmotic stress response pathway in the model organism *S. cerevisiae*. In this research, we will use all three simulation approaches offered by *Morven*, qualitative, semi-quantitative, and quantitative simulation, to study this stress response pathway. We will also perform relevant analysis based on the obtained simulation results to further demonstrate the suitability of this computational framework to the study of stress response pathways and system biology research in general.

The rest of this paper is organised as follows: the *Morven* framework is introduced in Section 2. In Section 3 the basic mechanism and existing models of the osmoregulation system of *S. cerevisiae* is presented. In Section 4 we focus on the qualitative modelling and simulation of the osmotic stress response pathway. Semi-quantitative and quantitative simulation of the pathway is detailed in Section 5. Finally conclusions and future work are presented in Section 6.

## 2 The *Morven* framework

The *Morven* framework is a qualitative reasoning (Kuipers, 1989; Forbus, 1997) system and first created by Coghill and Chantler (1994) and Coghill (1996). It was then further developed and re-implemented in Java, which resulted in *JMorven* (Bruce and Coghill, 2005; Bruce, 2007). To perform the research carried out in this paper we upgraded *JMorven* by enhancing its semi-quantitative simulator: the precision and reliability of the existing Taylor integration algorithm (Bruce, 2007) for semi-quantitative simulation was improved. Furthermore, the more precise Adams-bashforth integration algorithms (described later in this section) were also implemented for semi-quantitative simulation. In the rest of this paper, all descriptions and applications of *Morven* are based on this upgraded version of *JMorven*.

The development of *Morven* involved two of its qualitative reasoning predecessors, QSIM (Qualitative SIMulation) (Kuipers, 1986) and FuSim (Fuzzy Qualitative Simulation) (Shen and Leitch, 1993), as well as other relevant systems, such as Vector Envisionment (VE) (Morgan, 1988; Coghill, 1992) and Predictive Algorithm (PA) (Wiegand, 1991). However, as we focus on the applications of *Morven* in this paper, the implementation details and algorithm description of *Morven* will not be presented in this paper, and readers are directed to the above-mentioned references for more details. In the rest of this section, we will describe the model formalism and output of *Morven*.

### 2.1 An example biological system

**Fig. 1:**
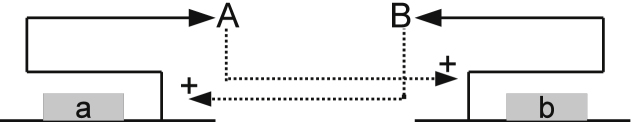
The two-gene regulatory network.

We first introduce a simple two-gene regulatory network as an example to illustrate the *Morven* model formalism. Fig. 1 shows the interactions of genes and proteins in this regulatory network. In this figure, genes a and b encode proteins A and B, respectively. The production of protein A activates the synthesis process of protein B, and vice versa. The dynamics of this network can be represented by the following two ODEs:

(1) $$x_{A}^{\prime }=f_{1}(x_{B})-g_{1}(x_{A})$$

(2) $$x_{B}^{\prime }=f_{2}(x_{A})-g_{2}(x_{B})$$

In the above two equations, *x*_*A*_ and *x*_*B*_ are concentrations of proteins A and B, separately. $x_{A}^{\prime }$ and $x_{B}^{\prime }$ are the rates of change of the protein concentrations. *f*_1_ and *f*_2_ are assumed to be monotonically increasing functions, which means the transcription rate of protein A (or B) increases (decreases) with the increase (decrease) of the concentration of protein B (or A). Within the allowed ranges of *x*_*A*_ and *x*_*B*_, $f_{1}^{\prime }$ and $f_{2}^{\prime }$ are both greater than zero. *g*_1_ and *g*_2_ are also monotonically increasing functions, which means the proteolysis rates of proteins increases (decreases) with the increase (decrease) of their own concentrations.

In particular, if we assume linear interaction relations we have the following model:

(3) $$x_{A}^{\prime }=k_{\mathrm{BA}}x_{B}-k_{A}x_{A}$$

(4) $$x_{B}^{\prime }=k_{\mathrm{AB}}x_{A}-k_{B}x_{B}$$

In the above two equations, *k*_*BA*_ and *k*_*AB*_ are both assumed to be positive constants, which means the transcription rate of protein A (or B) are proportional to the concentration of protein B (or A). *k*_*A*_ and *k*_*B*_ are also positive constants, which means the proteolysis rates of proteins are proportional to their own concentrations.

### 2.2 Qualitative modeling of *Morven*

A *Morven* qualitative model corresponding to Eqs. (1) and (2) is shown in Table 1. The details of this model will be explained in the rest of this section.

#### 2.2.1 Qualitative variables and quantity space

In *Morven* variables are in the form of vectors. The first element of the vector represents the magnitude of this variable; the other elements (if any) stand for higher derivatives. In Table 1 we see that for each constraint the derivative of a variable must be specified, for instance, (dt 0 *P*_*A*_) means the magnitude of *P*_*A*_ and (dt 1 *x*_*A*_) stands for the first derivative of *x*_*A*_.

In qualitative models, each element of the variable vector is associated with a *quantity space*, and the value of a variable can be only taken from its quantity space. A quantity space is composed of several quantity values, which are in the form of (*MinVal*,*MaxVal*) pairs and represent discrete intervals.^1^ *MinVal* and *MaxVal* define the range of this quantity value and satisfy *minVal* ≤ *MaxVal*. The values of *MinVal* and *MaxVal* could be real numbers, or positive and negative infinity (denoted as +∞ and −∞). The most commonly used quantity space is called the *signs* quantity space, which is composed of three values:

• *positive*, representing the interval (0, ∞)
• *negative*, representing the interval (−∞, 0)
• *zero*, representing (0, 0)

An example value of a *Morven* qualitative variable using the above signs quantity space is 〈*positive*, *negative*, *zero*〉, which means that the magnitude, first derivative, and second derivative are positive, negative, and zero, respectively. A variable taking this value means that this variable is positive and decreasing, but its decreasing rate (second derivative) remains steady.

#### 2.2.2 Qualitative models

From Table 1 we see that a *Morven* model is composed of several constraints, each of which has its corresponding mathematical relation. A *Morven* qualitative model is the conjunction of all its constraints, and these constraints are called *qualitative differential equations*. Constraints are distributed across different *differential planes*. The *0th* differential plane contains all constraints that could construct an equivalent quantitative model used for numerical simulation. Constraints in higher differential planes are obtained by differentiating the corresponding constraints in their immediately preceding differential planes. A *Morven* model can have as any number of differential planes as necessary.

There are two kinds of constraints in *Morven*: *function* constraints and *algebraic* constraints. A *function* constraint represents incomplete knowledge about the relation between two variables and only appears in qualitative models. A *function* constraint contains several mappings, which are empirically obtained and define the relation between its two variables. In the model presented in Table 1 constraints *C*1, *C*2, *C*4, and *C*5 are function constraints. If signs quantity spaces are used by all variables, there are generally three mappings for all these four function constraints:

• negative → negative
• zero → zero
• positive → positive

For ease of interpretation, in the rest of this paper a function constraint which includes and only includes the above mappings is called an increasing function and denoted as *M*_*inc*_. Similarly, a decreasing function *M*_*dec*_ is defined as a function constraint having the following mappings:

• negative → positive
• zero → zero
• positive → negative

*Algebraic* constraints describe algebraic relations of variables, such as *addition*, *subtraction*, *multiplication*, and *division*. Constraints *C*3 and *C*6 in Table 1 are examples of algebraic constraints.

Finally, in the model presented in Table 1, variables *P*_*A*_ and *D*_*A*_ are understood as the production and degradation components of protein A, respectively. *P*_*B*_ and *D*_*B*_ are defined analogously. Because of the algorithm implementation of *Morven*, complicated long mathematical equations have to be broken into two or three-place constraints.

### 2.3 Output of the qualitative simulation

In qualitative reasoning, a *qualitative state* is a complete assignment of values to all qualitative variables of the model, and a *qualitative behaviour* is a series of qualitative states linked by their *legal transitions*. If there is a legal transition from one qualitative state to another, the change in all variables in these two states must be *continuous* and *consistent*.

This is explained as follows: for any element of any variable in the model, the change of values in the two states must be (1) “adjacent” in the corresponding quantity space and (2) consistent with its immediate next derivative. For instance, a variable can change from 〈*positive*, *negative*〉 to 〈*positive*, *zero*〉 because this is a continuous and consistent change. However, the following three examples are not legal:

1. 〈*positive*, *negative*〉 → 〈*negative*, *negative*〉
2. 〈*positive*,*negative*〉 → 〈*positive*, *positive*〉
3. 〈*zero*, *negative*〉 → 〈*positive*, *negative*〉

In (1) the magnitude cannot change from *positive* to *negative* without passing *zero*. It is similar for the first derivative in (2). While in (3) although the change of the magnitude is continuous, it is not consistent with the first derivative, which indicates that the magnitude should decrease.

The output of a qualitative model can be an *envisionment*, a directed graph with nodes standing for possible qualitative states and edges representing transitions between qualitative states. The envisionment includes all possible *qualitative behaviours* of the model. In addition, a *total envisionment* is an envisionment in which exogenous variables can take all possible values. A *complete envisionment* is an evisionment in which each exogenous variable is assigned only one value. In Sections 4.1.2 and 4.2.2 we will see examples of such an *envisionment*.

### 2.4 Semi-quantitative and quantitative modelling in *Morven*

In *Morven* to perform semi-quantitative simulation, the model takes the same form as in qualitative simulation except for the following requirements:

• There are no *function* constraints in the model. Instead, they have to be replaced by equivalent algebraic constraint(s). Otherwise semi-quantitative and quantitative simulation cannot be performed because of lack of knowledge about the relations between variables.
• Although variables are associated with quantity spaces, during semi-quantitative simulation their initial values could be assigned any intervals and they may take any interval values during simulation.
• Parameters in a semi-quantitative model can also be intervals, which are specified by modellers.

As mentioned in Section 1 *Morven* can perform qualitative, semi-quantitative, and quantitative simulation using the same model. This is achieved by presenting the model used for semi-quantitative simulation to *Morven* but making different specifications to this model when performing the other two simulations, which is described as follows:

1. To perform qualitative simulation, variables are required to take values only from their quantity space.
2. To perform quantitative simulation, the initial values of all variables must be real numbers (interval = 0), and so are all model parameters.

From above we see that quantitative simulation in *Morven* is actually a special case of semi-quantitative simulation. Table 2 shows the *Morven* semi-quantitative model for the two-gene regulatory network based on Eqs. (3) and (4). In this model variables with the prefix *aux* are auxiliary variables. In Morven, auxiliary variables are not in the form of vectors but are scalar, and they are used to temporarily store intermediate calculation results.

### 2.5 Non-constructive interval simulation

*Morven* employs a non-constructive method (Wiegand, 1991; Kuipers, 1986) to perform the interval simulation, and this enables *Morven* to deal naturally with models described by DAEs (Differential Algebraic Equations), which are in the following form:

(5) $$F(y^{\prime },y,t)=0$$

In the above, *y* is a vector of variables depending on time *t*: *y*(*t*) = {*y*_1_(*t*), *y*_2_(*t*), …, *y*_*n*_(*t*)}. *y*′ is the derivative of *y*. *F* is a vector of functions in the form of *F* = (*F*_1_, *F*_2_, …, *F*_*n*_). It should be pointed out that in DAEs it is not always possible to solve the derivatives *y*′ in terms of *y*, as shown below:

(6) $$y^{\prime }=f(t,y)$$

In the above, *y* and *y*′ have the same meaning as Eq. (5), and *f* is a vector of functions in the form of *f* = (*f*_1_, *f*_2_, …, *f*_*n*_). Eq. (6) is required by most ODE solvers.

In order to perform simulation with *Morven*, Eq. (5) has to be broken into two-place or three-place constraints. The non-constructive simulation is briefly described as follows: for each time point (integration step), *Morven* updates the current interval values of all variables by iteratively scanning each constraint in the model and calculating new intervals based on these constraints using interval arithmetic. The iteration will stop when no more interval values can be updated. Then in the next time point, *Morven* calculates the interval values based on its derivatives at previous time points. The current version of *Morven* offers two kinds of integration approaches to interval simulation: (1) Taylor's method (Bruce, 2007), which uses the values of higher derivatives at the immediately prior time point and (2) two-step Adams–Bashforth method (its quantitative version is detailed by Shampine and Gordon (1975)), which uses the values of first derivatives at previous two time points. Both of these two approaches are the interval simulation versions of their numerical counterparts.

### 2.6 Output of semi-quantitative simulation

Given initial conditions of all variables, some of which are intervals, the semi-quantitative simulation results are composed of the time series data for all variables. For each variable at each time point, the upper bound and lower bound are given. These time series data can be visualised. Fig. 7 shows an example of such output.

## 3 The osmotic stress response pathway in yeast *S. cerevisiae*

In this section we give a brief introduction of the osmotic stress response pathway in yeast *S. cerevisiae*. We first present the basic mechanism of the stress response pathway. This is followed by a quick review of several existing models for the osmotic stress.

### 3.1 The basic mechanism of the pathway

**Fig. 2:**
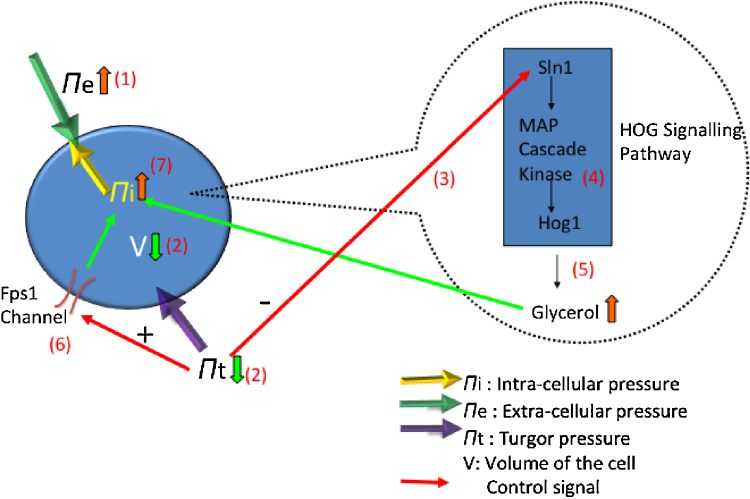
The osmotic stress response pathway.

Fig. 2 illustrates the osmotic stress response pathway. This figure is detailed as follows. (1) An increase of the concentration of sodium chloride in the cell medium leads to a sudden increase of the extra-cellular osmotic pressure (Π_*e*_). Yeast *S. cerevisiae* adapts to high extra-cellular osmotic pressure through the following process: (2) the cell volume *V* decreases to gain the intra-cellular pressure (Π_*i*_), and in the meantime the turgor pressure (Π_*t*_) decreases as well. (3) The loss of the turgor pressure triggers the High Osmolarity Glycerol (HOG) signalling pathway. In the HOG signalling pathway, first Sln1, a membrane-localised histidine kinase responsible for sensing the osmotic pressure change, is inactivated through dephosphorylation. (4) The inactivation of Sln1 causes the phosphorylation of Hog1 through the activation of a mitogen-activated protein (MAP) cascade kinase. (5) The activation of Hog1 (phosphorylated Hog1) enters the nucleus and accumulates there. The accumulated phosphorylated Hog1 in the nucleus leads to the transcription of two genes GPD1 and GPP2. These two genes are translated to proteins Gpd1 and Gpp2, which are involved in the production of the osmolyte glycerol. (Note in Fig. 2 detailed information about this step is not shown.) (6) Under high osmotic stress, the Fps1 channel closes to prevent the leakage of glycerol from inside the cell. The closing of the Fps1 channel can also be considered as a result of the turgor pressure loss. (7) Through the process of (3)–(5), together with (6), more and more glycerol will be accumulated in the cell, and this further increases the intra-cellular osmotic pressure, which in turn leads to the recovery of the cell volume and turgor pressure. Finally the cell adapts to the high extra-cellular osmotic pressure through the above described process.

### 3.2 Existing models

Several quantitative models have been proposed to model and analyse the osmotic stress response pathway. In this subsection, we described some representative modelling approaches.

#### 3.2.1 Detailed model

As mentioned in Section 1, the Klipp–Hohmann model (Klipp et al., 2005) describes the pathway in great detail by including as many as possible known species and components. To deal with the complexity, the model divides the system into several functional components: (1) the biophysical process, including the change of turgor pressure, cellular volume, and intra-cellular pressure; (2) the signalling pathway, including the Sln1 phosphorelay module and the MAP kinase cascade; (3) the gene expression module, which describes the transcription of genes GPD1, GPP2 and the translation of proteins Gpd1, Gpp2; (4) the metabolism component, which leads to the production of glycerol.

This kind of detailed modular model aims to permit a deep understanding of the pathway, quantitatively study the behaviours of each species and the interactions between components in the osmotic stress response system. Through numerical simulation of this detailed ODE model, the obtained time courses agree well with published experimental data.

#### 3.2.2 Simple model

Considering the complexity of the Klipp–Hohmann model, Gennemark et al. proposed a simple ODE model (Gennemark et al., 2006) for the osmotic stress response pathway in yeast. This simple model aims to capture the dynamics of the system economically by (1) treating some complicated yet not well-understood processes as black-box components and (2) only considering the most important species and processes of the system.

Compared to the detailed model, the simple model is easy to understand and analyse. In addition, the simple model is more reasonable because (1) in the osmoregulation system mechanisms of some processes, such as the Sln1 sensing process and the Hog1 signalling pathway, are not exactly known at a very detailed level and (2) only sparse experimental data are available.

#### 3.2.3 Minimal interaction model

Macia et al. (2009) proposed a minimal interaction model to study the high basal signal in the signalling pathway. The minimal model only considers the non-linear interaction between the sensor protein Sln1 and the key target protein Hog1, and reveals the role of the high basal signal in the osmoregulation. By assuming that the interactions between components follow the non-linear hill function, the response immediately after the stress is modelled by the following two differential equations:

(7) $$\frac{\mathrm{dSln}1}{\mathrm{dt}}=g(\mathrm{Sln}1,S),$$

(8) $$\frac{\mathrm{dHog}1p}{\mathrm{dt}}=f(\mathrm{Sln}1,\mathrm{Hog}1p).$$

In the above Hog1p stands for the phosphorylated Hog1, and functions *f* and *g* are non-linear hill functions. In this minimal model, the roles of all intermediate components between the Sln1 and Hog1 are implied in the non-linear hill functions. Nullcline analysis was performed on this model and possible feedbacks between components were hypothesised and analysed.

#### 3.2.4 Other models

Besides the above three models, there are also other models proposed to tackle various problems: Muzzey et al. (2009) proposed a model to study the perfect adaptation of nuclear enrichment of Hog1 to high osmotic pressure. Mettetal et al. (2008) built a model to analyse the frequency dependence of the signal transduction using periodic osmotic stresses. A common feature of these model is that they were built in such a way that specific mathematical analysis or simulation can be carried out.

## 4 Qualitative modelling and simulation

In this section, we present qualitative modelling approaches to model the osmoregulation system in yeast. First, we demonstrate that a qualitative model can be built from scratch based on the understanding of the biophysical process of osmoregulation. Second, we provide a solution by using an existing quantitative model to perform qualitative simulation of the whole process of the osmoregulation system.

### 4.1 A qualitative model for the biophysical process of the osmoregulation

We first propose a qualitative model for the biophysical process of the system. To simplify the problem, we focus on the biophysical response of the cell just after the stress, and we assume that the turgor pressure (Π_*t*_) is always bigger than zero. In this period the accumulation of glycerol is not significant enough to increase the intra-cellular pressure and thus can be ignored. We consider the extra-cellular pressure (Π_*e*_) as an exogenous variable and it has a linear increasing relation with the concentration of sodium chloride: extra-cellular pressure (in Osm) equals the concentration of sodium chloride (in molar) multiplied by 2 × 0.93, where 2 is the number of ions in NaCl and 0.93 is the osmotic coefficient of NaCl (Robinson and Stokes, 1959).

#### 4.1.1 Model description

We initially specify that all variables take the signs quantity space. First all constraints in the 0th differential plane of the model are given as follows:

(9) $$\mathrm{sub}\;(\mathrm{dt}\;0\;\Pi_{t})\;(\mathrm{dt}\;0\;\Pi_{o}\;)(\mathrm{dt}\;0\;\Pi_{e})$$

(10) $$\mathrm{sub}\;(\mathrm{dt}\;0\;\Pi_{\mathrm{Diff}})\;(\mathrm{dt}\;0\;\Pi_{i}\;)(\mathrm{dt}\;0\;\Pi_{o})$$

(11) $$M_{\mathrm{inc}}\;(\mathrm{dt}\;1\;V)\;(\mathrm{dt}\;0\;\Pi_{\mathrm{Diff}}\;)$$

(12) $$\mathrm{func}\;(\mathrm{dt}\;0\;\Pi_{i})\;(\mathrm{dt}\;0\;V\;)$$

(13) $$\mathrm{func}\;(\mathrm{dt}\;0\;\Pi_{t})\;(\mathrm{dt}\;0\;V\;)$$

Constraints in the 1st differential plane are obtained by differentiating the corresponding constraints in the 0th differential plane:

(14) $$\mathrm{sub}\;(\mathrm{dt}\;1\;\Pi_{t})\;(\mathrm{dt}\;1\;\Pi_{o}\;)(\mathrm{dt}\;1\;\Pi_{e})$$

(15) $$\mathrm{sub}\;(\mathrm{dt}\;1\;\Pi_{\mathrm{Diff}})\;(\mathrm{dt}\;1\;\Pi_{i}\;)(\mathrm{dt}\;1\;\Pi_{o})$$

(16) $$M_{\mathrm{inc}}\;(\mathrm{dt}\;2\;V)\;(\mathrm{dt}\;1\;\Pi_{\mathrm{Diff}}\;)$$

(17) $$M_{\mathrm{dec}}\;(\mathrm{dt}\;1\;\Pi_{i})\;(\mathrm{dt}\;1\;V\;)$$

(18) $$M_{\mathrm{inc}}\;(\mathrm{dt}\;1\;\Pi_{t})\;(\mathrm{dt}\;1\;V\;)$$

In the above *V* is the cell volume; Π_*i*_ is the intra-cellular pressure; Π_*o*_ is the sum of turgor pressure and extra-cellular pressure; Π_*Diff*_ is the pressure difference between Π_*i*_ and Π_*o*_. Under hyper-osmotic stress Π_*Diff*_ is non-positive and under hypo-osmotic stress it will be a non-negative value.

*M*_*inc*_ and *M*_*dec*_ are the function constraints described in Section 2.2.2. The first derivative of *V* has an increasing linear relation to Π_*Diff*_ if the area of the cell membrane is assumed to be constant (Gennemark et al., 2006):

(19) $$V^{\prime }=k\cdot \Pi_{\mathrm{Diff}}$$

In the above *k* is a positive constant. So we can write constraint (11). Differentiating Eq. (19) with respect to time *t* gives us the following equation:

(20) $$V^{\prime \prime }=k\cdot \Pi_{\mathrm{Diff}}^{\prime }$$

The above equation leads to constraint (16).

The function constraints in Constraints (12) and (13) include only one mapping: *positive* → *positive*. This is because it is known that the magnitudes of the cell volume, intra-cellular pressure, and turgor pressure are all positive values, and no further information about the relationships of their magnitudes is given. Constraints (17) and (18) indicate that the decrease (increase) in *V* will lead to the increase (decrease) in Π_*i*_ and decrease (increase) in Π_*t*_. As mentioned before the accumulation of glycerol is not considered in this biophysical model, the increase of the intra-cellular pressure is solely caused by the decrease in the cell volume.

#### 4.1.2 Simulation results

**Fig. 3:**
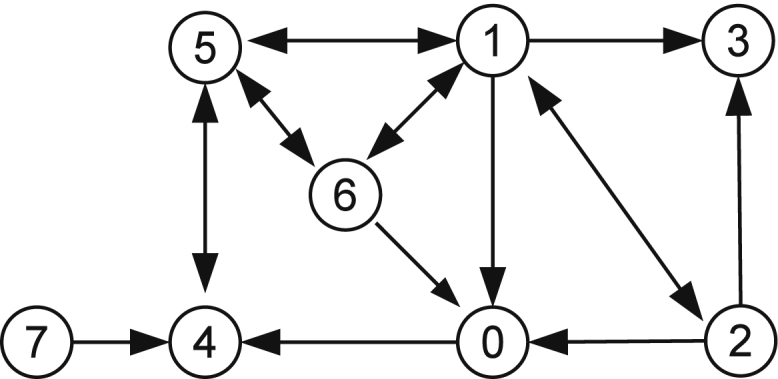
The total envisionment of the biophysical model.

The exogenous variable Π_*e*_ may take three different qualitative values: 〈*positive*, *zero*〉, 〈*positive*, *negative*〉, and 〈*positive*, *positive*〉. Fig. 3 shows the total envisionment (described in Section 2.3) when Π_*e*_ takes the above three qualitative values. The qualitative states in this envisionment are listed in Table 3. In this table, “+”, “0”, and “−” represent *positive*, *zero*, and *negative* in the signs quantity space, respectively.

The above envisionment predicted all possible qualitative states and their transitions of the biophysical system immediately after the stress. From the envisionment graph we see that there are only eight possible states. These states can be grouped into three subsets according to the values of the exogenous variable Π_*e*_: States 0 and 1 when Π_*e*_ is positive and steady; States 2 and 3 when Π_*e*_ is positive but decreasing; States 4–7 when Π_*e*_ is positive and increasing. In this sense there are three complete envisionment graphs, each of which contains one of the above three subsets of states. For instance, if Π_*e*_ is assumed to be always positive and steady, the complete envisionment will only include States 0 and 1, and there is only one transition from State 1 to State 0.

#### 4.1.3 Discussion

We can obtain some biophysical insights into the stress response pathway from the above generated envisionment graph. First, the simulation results indicate that starting from the same qualitative status, the biophysical system may demonstrate different stress response behaviours, each of which corresponds to a path extracted from the envisionment graph. For instance, if at the beginning all pressure variables remain constant, which means the system is at its steady state (State 0), and we slowly add a certain amount of salt into the cell media, the extra-cellular pressure Π_*e*_ will change from 〈+,0〉 to 〈+,+〉, and then change back to 〈+,0〉 again after we stop adding salt. Based on the graph, we see that the system may choose the behaviour 0 → 4 → 5 → 6 → 0 or behaviour 0 → 4 → 5 → 1 → 0. Which behaviour will be chosen depends on two factors: first, the characteristics of the system, for instance, the parameter value *k* in Eq. (19); second, the exact quantitative initial conditions, for instance, the exact quantitative value of the magnitude and first derivative of Π_*e*_. However, as at the qualitative level, there is no quantitative information for the above two factors, all possible behaviours have to be included into the envisionment.

Second, if throughout the biophysical process Π_*e*_ remains qualitatively the same (i.e., always increasing, decreasing, or constant), the system will undergo *at most* one qualitative change. This is explained as follows: as mentioned in Section 4.1.2, all states can be divided into three groups according different qualitative values of Π_*e*_: States 2 and 3; States 0 and 1; and States 4–7. Furthermore, if the second derivative of *V* is not considered, States 4–6 can be merged into one “super” state, denoted as State 4, and all other states remain the same. In this situation if the value of Π_*e*_ remains qualitatively the same, the system can only achieve two states, and there is only one single direction transition from one of these two states to another, that is, State 1 → State 0, State 2 → State 3, or State 7 → State 4. The above analysis comprehensively reveals the dynamics of the system when dealing with different values of Π_*e*_.

### 4.2 Qualitative simulation based on the Gennemark simple model

In this section we will show how *Morven* performs qualitative simulation from an existing quantitative model. We choose the Gennemark simple model (Gennemark et al., 2006), and the advantages of this model were briefly presented in Section 3.2.2. Although both the Gennemark and Klipp–Hohmann models can describe the complete stress response process, the Gennemark model is more suitable for qualitative simulation because of its simplicity and ability to capture all important dynamics. In particular, the reasons for not choosing the Klipp–Hohmann model for simulation are as follows: first, the simulation results obtained from such a complex model are not easy to analyse. Many variables in the Klipp–Hohmann model cannot be measured at all, and many parameters are taken estimated values. Simulating a qualitative model converted from such kind of model will result in a huge number of qualitative states, many of which are meaningless in the sense that many variables in these states cannot be verified, and these states do not offer any insight and make the simulation results confusing and hard to analyse. Second, the Klipp–Hohmann model is hard to simulate qualitatively. The computational cost of qualitative simulation increases exponentially with the number of qualitative constraints in the model. Simulating such a complex model will be computationally intractable.

It should be pointed out that the Gennemark model is not a simplified version of the Klipp–Hohmann model in the sense that it has different modelling scope and employs different modelling techniques.

#### 4.2.1 Model description

The conversion of the Gennemark ODE model to a *Morven* model is straightforward: (1) each ODE is rewritten as *Morven* constraints. (2) The parameters in the model are treated as exogenous variables because they are not determined by variables within the system. Unless specified, parameter values are taken from the original quantitative model. (3) All variables are associated with the signs quantity space.

To simplify the conversion, we only consider models in the 0th differential plane. In addition, we assume the turgor pressure is always positive for ease of exposition. (When turgor pressure becomes zero, the system behaviour will be governed by a different model. For this paper we focus on single model simulation.)

The converted model is shown in Table 4. In this model, variables and parameters take the same names as in the original quantitative model, as shown in Tables 5 and 6. Variables starting with “aux” are auxiliary variables, which have the same function as those in the model shown in Table 2. Operators *add*, *sub*, *mul*, and *div* stand for addition, subtraction, multiplication, and division, respectively.

#### 4.2.2 Simulation results

To perform qualitative simulation on the model presented in Table 4, we specify that all variables take the signs quantity space. The magnitudes of all variables are positive except that the magnitude of ${\tilde{u}}_{\mathrm{HOG}}$ could be zero.

**Fig. 4:**
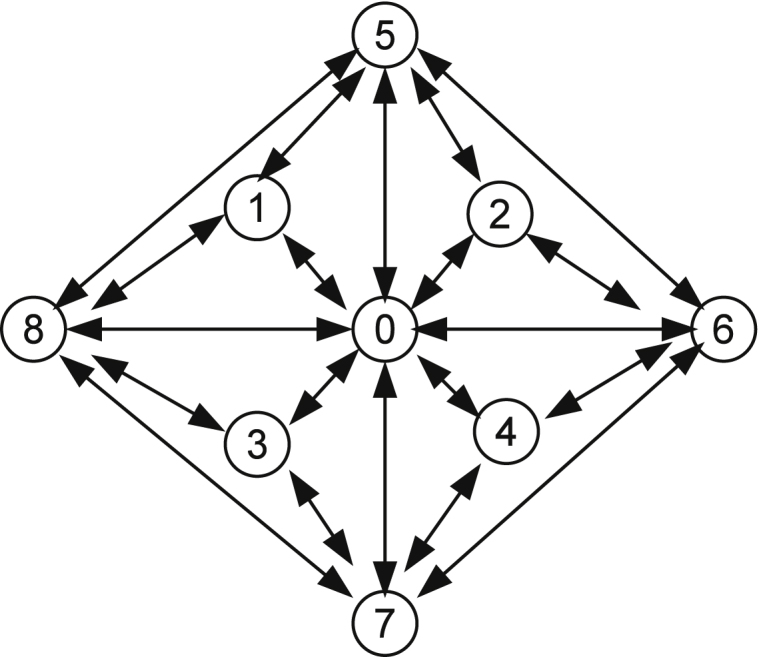
The envisionment graph containing variables *V* and *Gly*.

We first perform the total envisionment which only includes variables *V* and *Gly* because these two variables are easier to measure than others. Considering that each variable can take three possible values: 〈*positive*, *zero*〉, 〈*positive*, *negative*〉, and 〈*positive*, *positive*〉, there are 9 possible states. The obtained envisionment graph shown in Fig. 4 contains all these 9 states, which are listed in Table 7. The obtained results clearly shows that qualitative simulation can be performed on a model converted from an existing quantitative model. To obtain more precise prediction, more variables can be included and additional conditions can be specified.

Similarly, we include different combinations of variables in the envisionment and calculate how many states the resulting envisionment graph can rule out. For instance, if we include *V*, *Gly*, and *Gly*_*e*_ there are 27 possible states and the envisionment contains 18 of them. This means the other 9 states can never be achieved by the system. Table 8 lists the results of all experiments. From this table we can see that with the inclusion of more variables more impossible states will be identified. For instance, the last experiment tells us that if all state variables are considered 96 out of 135 states will be excluded from the evisionment graph.

### 4.3 Conclusions and discussion

In this section, we first demonstrated that a *Morven* qualitative model can be built from scratch based on an initial understanding about the biophysical process of the osmoregulation system. The resulting envisionment graph presents a global picture of the dynamics of the stress response achieved by the biophysical process, which can help us gain a deeper understanding about the mechanisms underlying the system. This kind of qualitative analysis is very important at the early stage of the modelling process when a quantitative model is not available due to insufficient knowledge and data. An analysis on the results obtained from the qualitative simulation of the biophysical model show that even without quantitative information about the pathway, biophysical insights can still be gained.

In the second part of this section, we demonstrated that *Morven* can perform qualitative simulation on a model converted from an established quantitative model. This kind of qualitative simulation is a complementary approach to quantitative analysis, and can reveal the qualitative dynamical behaviours of the system, which may not be easily captured by quantitative simulation. More importantly, qualitative simulation can predict possible states and exclude impossible ones, which will facilitate further quantitative analysis and suggest new biological experiments to verify the predicted behaviours.

Both modelling examples presented in this section clearly show that qualitative modelling and simulation performed with *Morven* can help better understand the stress response mechanisms of *S. cerevisiae* either as an independent tool or a complementary approach. In addition, we point out that our qualitative modelling and simulation approach can further help the hypothesis formation and testing when studying the stress response pathway in other species. For instance, the osmotic stress response pathway in *Candida albicans* is less known compared to that in *S. cerevisiae*. From biological experiments we know that both stress response pathways share similarities and there are also differences (Quinn and Brown, 2007). For instance, both species accumulate glycerol in response to osmotic stress. However, in *C. albicans* no Fps1 homolog has been found until now, even though a similar function to the Fps1 channel as in *S. cerevisiae* has been hypothesised. To build a stress response model for *C. albicans*, it is essential to form and test new hypotheses based on the well-studied stress response model of *S. cerevisiae*. At the qualitative level it is easier, and more reasonable to perform such hypothesis formation and testing tasks due to the fact that limited knowledge is known and only sparse experimental data are available for the study of stress response mechanisms of *C. albicans* at the current stage. The above considerations also suggest future directions of our qualitative modelling and simulation approaches.

## 5 Semi-quantitative and quantitative simulation

In Section 2.5 the semi-quantitative and quantitative simulation algorithms employed by *Morven* were briefly introduced. In this section we use *Morven* to perform semi-quantitative and quantitative simulation for the osmotic stress response pathway. The simulation results will be presented and some discussion about the results will be given.

### 5.1 Model description

The model presented in Table 4 is chosen for the semi-quantitative and quantitative simulation. This also demonstrates that the same *Morven* model can be used for all three kinds of simulation.

### 5.2 Quantitative simulation

To perform quantitative simulation, the initial conditions of all variables and values of all parameters of the model should be real numbers rather than intervals. To compare the simulation results with those obtained from conventional numerical simulation, we re-implemented the Gennemark quantitative model in *Python*^2^ using the scientific computing package *SciPy*^3^, in which the ODE integration function *odeint* is used.

**Fig. 5:**
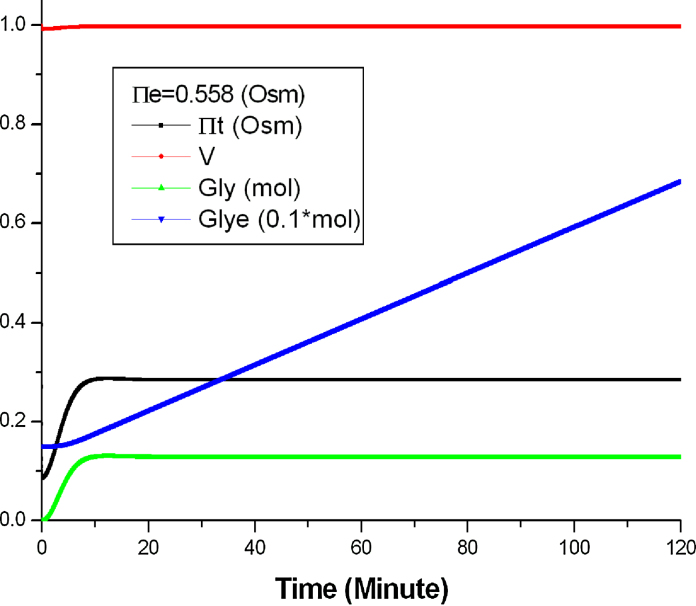
The quantitative simulation with *Morven* when Π_*e*_ = 0.558 Osm.

**Fig. 6:**
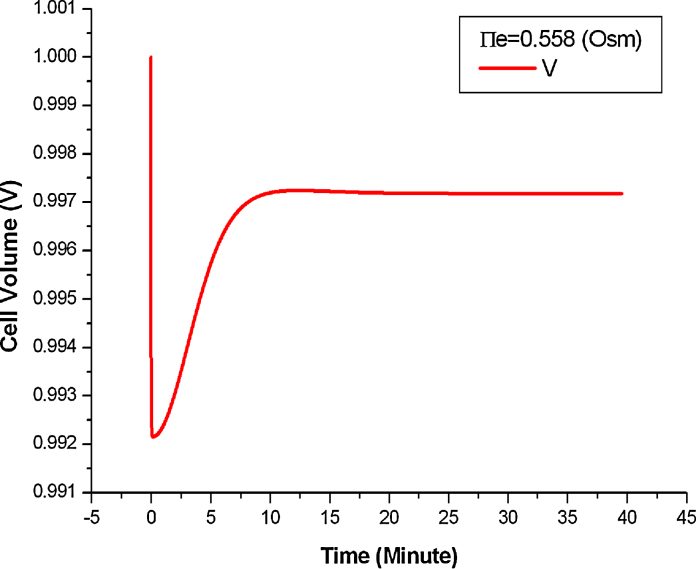
The quantitative simulation of the cell volume (*V*) with *Morven* when Π_*e*_ = 0.558 Osm.

The parameter values used for quantitative and semi-quantitative simulation are the same as those in the original Gennemark model, as listed in Table 6. Fig. 5 shows the quantitative simulation performed by *Morven* when Π_*e*_ = 0.558 Osm. Fig. 6 shows the change of the cell volume during the stress response process as this cannot be easily see from Fig. 5. The simulation results were further verified by numerical simulation using *Python* + *Scipy*. It is shown that results obtained from *Morven* are the same as those from conventional numerical simulation methods.

### 5.3 Semi-quantitative simulation

We aim to perform three kinds of semi-quantitative simulation: (1) the initial values of some variables are intervals; (2) the values of some parameters are intervals; (3) both initial conditions and parameters are intervals. Unless stated, the values of other parameters and variables are the same as those taken in the Gennemark model.

**Fig. 7:**
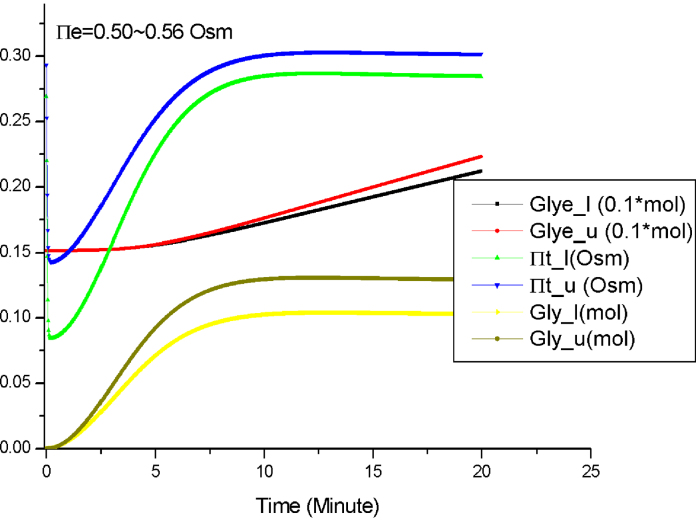
The semi-quantitative simulation by *Morven* when Π_*e*_ = 0.50–0.56 Osm.

First, we perform the simulation when the initial values are intervals. We set the initial value of Π_*e*_ to an interval: 0.50–0.56 Osm. Fig. 7 shows the obtained simulation results. In the legend of this figure the suffixes “_l” and “_u” indicate the lower and upper bounds of a variable, respectively. From this figure we see that the general curvature of the variable values agree with that from numerical simulation. However, for the value of a variable at each time point, their upper and lower bounds are estimated. This allows us to infer intuitively the dynamics of the pathway model at a semi-quantitative level.

**Fig. 8:**
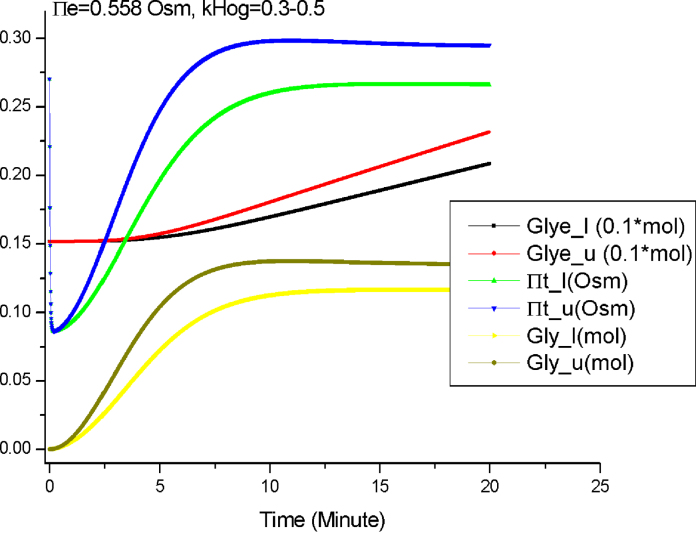
The semi-quantitative simulation by *Morven* when Π_*e*_ = 0.558 Osm and *k*_*HOG*_ = 0.3–0.5.

Second, we perform the simulation when some of the parameter values are intervals. We set the value of *k*_*HOG*_ to be an interval (0.3–0.5). Fig. 8 shows the simulation results.

**Fig. 9:**
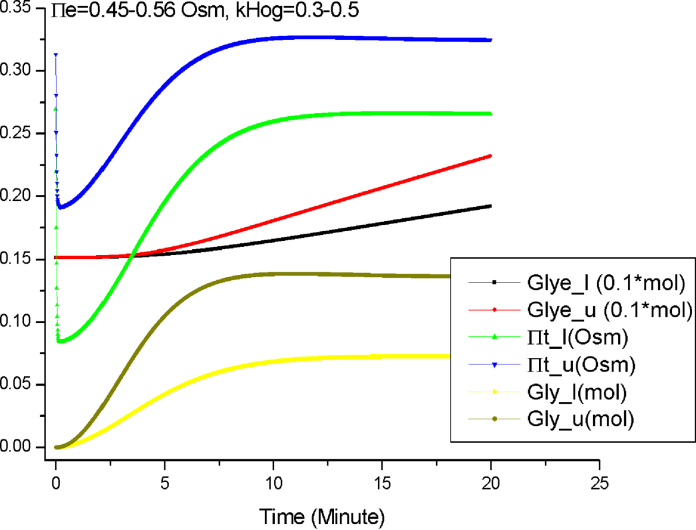
The semi-quantitative simulation by *Morven* when Π_*e*_ = 0.45–0.56 Osm and *k*_*HOG*_ = 0.3–0.5.

Third, we perform the simulation when both the initial conditions and the parameter values are intervals: Π_*e*_ = 0.45–0.56 Osm and *k*_*HOG*_ = 0.3–0.5. Fig. 9 shows the simulation results.

### 5.4 Discussion

In this section we presented the semi-quantitative and quantitative simulation of the osmotic stress response pathway performed by *Morven*. The quantitative simulation performed by *Morven* can obtain the same results as those from conventional numerical simulator. In addition, as mentioned in Section 2.5, *Morven* has the ability to simulate models described by any general DAEs, which have to be converted to ODEs in order for conventional numerical simulators to use them.

Results obtained from the semi-quantitative simulation provide a more robust estimation when initial conditions and parameter values are intervals. More importantly, the semi-quantitative simulation reveals dynamic behaviours that cannot easily be observed by quantitative or qualitative simulation.

From Fig. 7 we can see that at the initial stage of the stress response (the first 2.5 min), Π_*t*_ is very sensitive to the change of Π_*e*_ while *Gly* is not. This can be explained as follows: at the beginning of the stress response, the biophysical process takes the dominant role of the stress response, and Π_*t*_ responses very quickly upon the change of Π_*e*_. On the other hand, the accumulation of glycerol involves several biochemical reactions, which is a slower process compared to the fast biophysical response.

Fig. 8 tells us that different values of proportional control constant *k*_*HOG*_ result in different final steady values of Π_*t*_ and *Gly*. In addition, the influence of *k*_*HOG*_ on Π_*t*_ and *Gly* gradually increases over time: the intervals of these two variables first increase over time, and then remain unchanged. These results can help us better understand the mechanism of the proportional control and how it regulates the stress response pathway. More importantly, these intuitive results can provide directions for further mathematical analysis and experimental validation.

Finally Fig. 9 shows that when both the initial condition and parameter value are intervals, broader intervals will be obtained. This is not surprising because two influence factors combined together lead to more uncertain behaviours of the system.

The results presented in Fig. 7–9 show that the semi-quantitative simulation is an effective method capable of capturing important behaviours about the osmotic stress response pathway. It bridges the gap between qualitative analysis and quantitative analysis, and thus enables *Morven* to perform a complete spectrum of simulation. Based on these semi-quantitative simulation results, further mathematical analysis, such as the parameter sensitivity analysis, can be carried out. Finally the findings through semi-quantitative simulation and additional analysis based on these findings will suggest future biological experiments.

## 6 Discussions, conclusions, and future work

In this paper we employ the *Morven* framework to simulate the osmoregulation system of yeast at qualitative, semi-quantitative, and quantitative levels. Simulation results have shown that *Morven* can capture dynamics of the stress response pathway at different levels of abstraction, and these results can provide suggestions for further mathematical analysis and experimental validation.

Traditional quantitative modelling and simulation approaches alone are not enough to deal with modelling challenges existing in systems biology, and this necessitates the use of novel modelling approaches, such as the *Morven* framework. Even in situations where quantitative modelling approaches can be used *Morven* can still be a complementary modelling approach, although *Morven* itself offers quantitative simulation module. In addition, *Morven* can use the same model to perform all three kinds of simulation and has the ability to deal with DAEs by means of non-constructive algorithms. All of the above mentioned features of *Morven* make the modelling work more effective, especially when dealing with complex biological systems.

It should be pointed out that at the current stage we focus on the development of simulation tools for studying the stress response pathway. We acknowledge that it is essential to make use of biological experimental data to verify proposed models, and one solution is to utilise *Morven* in model inference systems as a verification component: *Morven* will simulate models generated from the model inference system and the simulation results will be compared with experimental data to evaluate the fitness of these models.

There has already been some work done towards the above proposed solution. For instance, in our previous work the qualitative simulator of *Morven* has been used as a model verification component in *QML-Morven* (Pang and Coghill, 2010a), a model learning system which automatically infers *Morven* qualitative models from given data and background knowledge, to study the detoxification pathway of Methylglyoxal (Pang and Coghill, 2011b) as well as the compartmental models (Pang and Coghill, 2010, 2011).

In the future, based on existing work on semi-quantitative model learning (Kay et al., 2000; Vatcheva et al., 2005), we aim to further develop *QML-Morven*, and make it able to infer models at both qualitative and semi-quantitative levels. The extended version of *QML-Morven* will utilise both the qualitative and semi-quantitative simulators of *Morven* to evaluate candidate models. This new version of *QML-Morven* will be used to study the osmoregulation system of yeast. By modelling and learning both qualitative and semi-quantitative models of the osmotic stress response pathway, we can effectively identify the models of the osmoregulation system from available experimental data and knowledge, and gain more understanding of the underlying mechanisms of the osmoregulation system at varying abstraction levels.

## Figures and Tables

**Table 1:** The *Morven* model for the two-gene network.

Constraint ID	*Morven* qualitative model	Mathematical relation
*0th differential plane*		
*C1*	func (dt 0 *P*_*A*_)(dt 0 *x*_*B*_)	*P*_*A*_ = *f*_1_(*x*_*B*_)
*C2*	func (dt 0 *D*_*A*_) (dt 0 *x*_*A*_)	*D*_*A*_ = *g*_1_(*x*_*A*_)
*C3*	sub (dt 1 *x*_*A*_) (dt 0 *P*_*A*_) (dt 0 *D*_*A*_)	$x_{A}^{\prime }=P_{A}-D_{A}$
*C4*	func (dt 0 *P*_*B*_) (dt 0 *x*_*A*_)	*P*_*B*_ = *f*_2_(*x*_*A*_)
*C5*	func (dt 0 *D*_*B*_) (dt 0 *x*_*B*_)	*D*_*B*_ = *g*_2_(*x*_*B*_)
*C6*	sub (dt 1 *x*_*B*_) (dt 0 *P*_*B*_) (dt 0 *D*_*B*_)	$x_{B}^{\prime }=P_{B}-D_{B}$

*1st differential plane*		
*C7*	func (dt 1 *P*_*A*_)(dt 1 *x*_*B*_)	$P_{A}^{\prime }=f_{1}^{\prime }(x_{B}^{\prime })$
*C8*	func (dt 1 *D*_*A*_) (dt 1 *x*_*A*_)	$D_{A}^{\prime }=g_{1}^{\prime }(x_{A})$
*C9*	sub (dt 2 *x*_*A*_) (dt 1 *P*_*A*_) (dt 1 *D*_*A*_)	$x_{A}^{\prime \prime }=P_{A}^{\prime }-D_{A}^{\prime }$
*C10*	func (dt 1 *P*_*B*_) (dt 1 *x*_*A*_)	$P_{B}^{\prime }=f_{2}^{\prime }(x_{A}^{\prime })$
*C11*	func (dt 1 *D*_*B*_) (dt 1 *x*_*B*_)	$D_{B}^{\prime }=g_{2}^{\prime }(x_{B}^{\prime })$
*C12*	sub (dt 2 *x*_*B*_) (dt 1 *P*_*B*_) (dt 1 *D*_*B*_)	$x_{B}^{\prime \prime }=P_{B}^{\prime }-D_{B}^{\prime }$

**Table 2:** The *Morven* semi-quantitative model for the two-gene network.

ID	*Morven* model	Mathematical relation
*C1*	mul (dt 0 *aux*1) (dt 0 *k*_*BA*_) (dt 0 *x*_*B*_)	*aux*1 = *k*_*BA*_ × *x*_*B*_
*C2*	mul (dt 0 *aux*2) (dt 0 *k*_*A*_) (dt 0 *x*_*A*_)	*aux*2 = *k*_*A*_ × *x*_*A*_
*C3*	sub (dt 1 *x*_*A*_) (dt 0 *aux*1) (dt 0 *aux*2)	$x_{A}^{\prime }=\mathrm{aux}1-\mathrm{aux}2$
*C4*	mul (dt 0 *aux*3) (dt 0 *k*_*BA*_) (dt 0 *x*_*A*_)	*aux*3 = *k*_*BA*_ × *x*_*A*_
*C5*	mul (dt 0 *aux*4) (dt 0 *k*_*B*_) (dt 0 *x*_*B*_)	*aux*4 = *k*_*B*_ × *x*_*B*_
*C6*	sub (dt 1 *x*_*B*_) (dt 0 *aux*3) (dt 0 *aux*4)	$x_{B}^{\prime }=\mathrm{aux}3-\mathrm{aux}4$

**Table 3:** Qualitative states in Fig. 3.

State	V	Π_*i*_	Π_*e*_	Π_*t*_
0	〈+,0,0〉	〈+,0〉	〈+,0〉	〈+,0〉
1	〈+,−,+〉	〈+,+〉	〈+,0〉	〈+,−〉
2	〈+,−,+〉	〈+,+〉	〈+,−〉	〈+,−〉
3	〈+,0,+〉	〈+,0〉	〈+,−〉	〈+,0〉
4	〈+,−,−〉	〈+,+〉	〈+,+〉	〈+,−〉
5	〈+,−,0〉	〈+,+〉	〈+,+〉	〈+,−〉
6	〈+,−,+〉	〈+,+〉	〈+,+〉	〈+,−〉
7	〈+,0,−〉	〈+,0〉	〈+,+〉	〈+,0〉

**Table 4:** The *Morven* model converted from the Gennemark simple model.

ID	Constraint	Mathematical relation
C0	add(dt 0 *aux*01)(dt 0 Π_*e*_)(dt 0 Π_*t*_)	*aux*01 = Π_*e*_ + Π_*t*_
C1	sub(dt 0 *aux*02)(dt 0 Π_*i*_)(dt 0 *aux*01)	*aux*02 = Π_*i*_ − *aux*01
C2	mul(dt 1 V)(dt 0 *k*_*p*1_)(dt 0 *aux*02)	*V*′ = *k*_*p*1_*aux*02
C3	add(dt 0 *aux*03)(dt 0 n)(dt 0 Gly)	*aux*03 = *n* + *Gly*
C4	sub(dt 0 *aux*04)(dt 0 V)(dt 0 *V*_*b*_)	*aux*04 = *V* − *V*_*b*_
C5	div(dt 0 Π_*i*_)(dt 0 *aux*03)(dt 0 *aux*04)	Π_*i*_ = *aux*03/*aux*04
C6	sub(dt 0 *aux*05)(dt 0 V)(dt 0 *V*^Π_*t*_=0^)	*aux*05 = *V* − *V*^Π_*t*_=0^
C7	mul(dt 0 *aux*06)(dt 0 Π_*t*_(0))(dt 0 *aux*05)	*aux*06 = Π_*t*_(0)*aux*05
C8	sub(dt 0 *aux*07)(dt 0 V(0))(dt 0 Π_*t*_(0))	*aux*07 = *V*(0) − Π_*t*_(0)
C9	div(dt 0 Π_*t*_)(dt 0 *aux*06)(dt 0 *aux*07)	Π_*t*_ = *aux*06/*aux*07
C10	mul(dt 0 *aux*08)(dt 0 *k*_*p*2_) (dt 0 Π_*t*_)	*aux*08 = *k*_*p*2_Π_*t*_
C11	div(dt 0 *u*_*fps*1_)(dt 0 *aux*08) (dt 0 Π_*t*_(0))	*u*_*fps*1_ = *aux*08/Π_*t*_(0)
C12	div(dt 0 *aux*09)(dt 0 Gly) (dt 0 *aux*04)	*aux*09 = *Gly*/*aux*04
C13	div(dt 0 *aux*10)(dt 0 *Gly*_*e*_) (dt 0 *V*_*e*_)	*aux*10 = *Gly*_*e*_/*V*_*e*_
C14	sub(dt 0 *aux*11)(dt 0 *aux*09) (dt 0 *aux*10)	*aux*11 = *aux*09 − *aux*10
C15	mul(dt 1 *Gly*_*e*_) (dt 0 *u*_*fps*1_) (dt 0 *aux*11)	${\mathrm{Gly}}_{e}^{\prime }=u_{\mathrm{fps}1}\mathrm{aux}11$
C16	sub(dt 0 *aux*12)(dt 0 Π_*t*_(0))(dt 0 Π_*t*_)	*aux*12 = Π_*t*_(0) − Π_*t*_
C17	mul(dt 0 *u*_*HOG*_)(dt 0 *k*_*HOG*_) (dt 0 *aux*12)	*u*_*HOG*_ = *k*_*HOG*_*aux*12
C18	sub(dt 0 *aux*13)(dt 0 *u*_*HOG*_) (dt 0 ${\tilde{u}}_{\mathrm{HOG}}$)	$\mathrm{aux}13=u_{\mathrm{HOG}}-{\tilde{u}}_{\mathrm{HOG}}$
C19	div(dt 1 ${\tilde{u}}_{\mathrm{HOG}}$)(dt 0 *aux*13) (dt 0 *t*_*d*_)	${\tilde{u}}_{\mathrm{HOG}}^{\prime }=\mathrm{aux}13/t_{d}$
C20	sub(dt 1 Gly) (dt 0 ${\tilde{u}}_{\mathrm{HOG}}$) (dt 1 *Gly*_*e*_)	${\mathrm{Gly}}^{\prime }={\tilde{u}}_{\mathrm{HOG}}-{\mathrm{Gly}}_{e}^{\prime }$

**Table 5:** Variables in the Gennemark model.

Name	Meaning
V	Cell volume
Π_*i*_	Intra-cellular osmotic pressure
Π_*t*_	Turgor pressure
Π_*e*_	Extra-cellular osmotic pressure
Gly	Intra-cellular glycerol
*Gly*_*e*_	Extra-cellular glycerol
*u*_*HOG*_	The control function of the HOG1 pathway
${\tilde{u}}_{\mathrm{HOG}}$	The delayed variable of *u*_*HOG*_

**Table 6:** Parameters in the Gennemark model.

Name	Meaning	Value
*k*_*p*1_	Water permeability coefficient times cell membrane area (Osm^−1^)	1.000
*n*	No. of other osmotically active compounds (mol)	0.402
*V*_*b*_	Non-osmotic volume of the cell	0.368
*V*^Π_*t*_=0^	Cell volume when Π_*t*_=0	0.990
Π_*t*_(0)	Initial value of Π_*t*_	0.396
V(0)	Initial cell volume	1.000
*k*_*p*2_	Glycerol permeability coefficient in a completely open Fps1 channel	0.316
*V*_*e*_	The fraction of the extra-cellular	4786.779
	volume belonging to each cell	
*k*_*HOG*_	Proportional control constant (Osm^−1^)	0.416
*t*_*d*_	Time delay (min)	8.611

**Table 7:** States of the envisionment graph shown in Fig. 4.

State ID	*V*	*Gly*
0	〈+,0〉	〈+,0〉
1	〈+,+〉	〈+,+〉
2	〈+,+〉	〈+,−〉
3	〈+,−〉	〈+,+〉
4	〈+,−〉	〈+,−〉
5	〈+,+〉	〈+,0〉
6	〈+,0〉	〈+,−〉
7	〈+,−〉	〈+,0〉
8	〈+,0〉	〈+,+〉

**Table 8:** States predicted by different envisionment graphs.

Variables	Predicted states	Impossible states
*V*, *Gly*	9	0
*V*, *Gly*, *Gly*_*e*_	18	9
*V*, *Gly*, ${\tilde{u}}_{\mathrm{HOG}}$	27	18
*V*, *Gly*, *Gly*_*e*_, ${\tilde{u}}_{\mathrm{HOG}}$	39	96

## Footnotes

1 As in FuSim, a quantity value in *Morven* is actually a fuzzy number composed of four tuples (Bruce and Coghill, 2005). In this paper, as we do not use fuzzy simulation, for ease of intepretation, we say that a quantity value is in the form of discrete intervals.

2 http://www.python.org.

3 http://www.scipy.org.
