# Supplementary material for: Qualitative, semi-quantitative, and quantitative simulation of the osmoregulation system in yeast
Source: Biosystems. 2015 May;131:40–50. doi: 10.1016/j.biosystems.2015.04.003 (PMC4441110; doi:10.1016/j.biosystems.2015.04.003)
Supplement: Supplementary file 1 [file mmc1.pdf]

Supplementary Material

Wei Pang and George M. Coghill

May 15, 2014

1 The JMorven Program File and User Manual

The JMorven program file *JMorvenV1.1a.jar* as well as some example model files are available for download at `https://sites.google.com/site/jmorven/`. A comprehensive user manual for JMorven, named *MorvenManual.pdf*, is also available for download at the same website.

2 Model Files for Reproducing Simulation Results

To reproduce the simulation results described in the manuscript, the user is suggested to first read the JMorven user manual. The relevant files used for simulation with JMorven are compressed into a zip file named *Data.zip*, which can be downloaded from `https://sites.google.com/site/jmorven/`. After decompressing this zip file, a folder called *Data* will be created, and all relevant files for simulation are stored in this folder. You can load these files into JMorven and perform simulation according to the user manual.

2.1 The two-gene regulatory network

The two-gene regulatory network is shown in Figure 1 in the manuscript, and its JMorven model is given in Table 1. The model file for simulating this system is *TwoGen_SimpleQS.txt*, and the quantity space file is *simpleQS.txt*. Both of these two files are in the subfolder *Data/twoGenNetwork*. To perform simulation (total and complete envisionment), simply load these two files into JMorven and follow the instruction from the user manual.

2.2 The qualitative model for the biophysical process of the osmoregulation

The qualitative model for the biophysical process of the osmoregulation is described in Section 4.1 of the manuscript, and the model and quantity space files are *BioPhyOsmotic.txt* and *simpleQS.txt*, both of which are located in the subfolder *Data/OsmoticStressModel/QulitativeModel/BiophysicalModel*. To reproduce the results as shown in Figure 3 of the

manuscript, load these two files and perform the *total envisionment* according to the user manual.

2.3 Qualitative simulation for the Gennemark simple model

To reproduce the complete envisionment shown in Figure 4 and Table 7 of the manuscript, we should load the model file *OsmoticModel_V&Gly.txt* and the quantity space file *OsmoticsimpleQS.txt*, both of which can be found in the subfolder *Data/OsmoticStressModel/QulitativeModel/CompleteModel*.

2.4 Quantitative and semi-quantitative simulation for the Gennemark simple model

To reproduce all the simulation results presented in Section 5 of the manuscript, we should use the model file *OsmoticModel.txt* and quantity space file *OsmoticQSpaces.txt*, both of which can be found in the subfolder *Data/OsmoticStressModel/SemiQuantitativeModel*. It is noted that the model and quantity space files are basically the same as those used for qualitative simulation. The only difference is that in qualitative simulation we may have different "printout" variables (variables to be shown in the envisionment), which are specified in the last line of the model file.

2.4.1 Quantitive simulation

To perform the quantitative simulation as shown in Figures 5 and 6 of the manuscript, after loading the model and quantity space files we should click the button "Select SemiQ Initial State" and load the initial values file *Initial_StateSemiQPe=0.558.txt*, which is in the same subfolder as the model file (*Data/OsmoticStressModel/SemiQuantitativeModel*). Parse the input files by clicking the "Parse Input Files" button. Then click "Simulate SemiQ" button, and in the popup windows set the time step size to be 0.01, the *length of time to simulate over* to be 120 (to make a quick simulation we can specify a shorter time period, say, 10), the simulation mode to be *Basic Interval Simulation*, and the integration mode to be *Taylor Method*. Perform the quantitative simulation and see the results obtained.

2.4.2 Semi-quantitative simulation I

To perform the semi-quantitative simulation as shown in Figure 7, we use the initial values file *Initial_SemiQPe=0.50-0.56.txt*, which can be found in the folder *Data/OsmoticStressModel/SemiQuantitativeModel*. The rest of the settings are the same as the previous quantitative simulation (time step=0.01, simulation time=10, integration mode: taylor method) apart from the simulation mode. The following simulation modes can be selected for the simulation: "Extreme Points Simulation", "Monte Carlo Point Simulation", and "Extreme Points & Monte Carlo Points Simulation", which combines both extreme point and Monte

Carlo simulation. If "Monte Carlo Point Simulation" or "Extreme Points & Monte Carlo Points Simulation" is selected, the number of points used for the Monte Carlo simulation should be provided: the larger this number is, the slower and more precise the simulation becomes.

2.4.3 Semi-quantitative simulation II

To perform the semi-quantitive simulation as shown in Figures 8 and 9, select the initial value files *Initial_StateSemiQPe=0.558kHog=0.3-0.5.txt* and *Initial_SemiQPe=0.45-0.56Khog0.3-0.5.txt*, respectively. These two files can be found in the folder *Data/OsmoticStressModel/ SemiQuantitativeModel*. Other settings are the same as those used for the previous semi-quantitative simulation.
