# Supplementary material for: Qualitative, semi-quantitative, and quantitative simulation of the osmoregulation system in yeast
Source: Biosystems. 2015 May;131:40–50. doi: 10.1016/j.biosystems.2015.04.003 (PMC4441110; doi:10.1016/j.biosystems.2015.04.003)
Supplement: Supplementary file 2 [file mmc2.pdf]

JMorven User Guide

Wei Pang and George M. Coghill

July 31, 2013

Contents

1 Introduction **4**

2 Installation **4**
2.1 JDK or JRE 4
2.2 Running JMorven 4

3 Introduction to the JMorven Main Window **5**

4 Qualitative Simulation **6**
4.1 Load Model File 6
4.2 Load Quantity Spaces File 6
4.3 Parse Input Files 6
4.4 Total Envisionment 7
4.5 Complete Envisionment 9
4.6 Stepwise Qualitative Simulation 11
4.6.1 Load Model File and Quantity Spaces File 11
4.6.2 Complete Envisionment 11
4.6.3 Choose Initial State 11
4.6.4 Perform the Simulation 11

5 Semi-quantitative Simulation **12**
5.1 Load Model File and Quantity Spaces File 12
5.2 Perform Simulation Using a Predefined Initial Values File 12
5.3 Perform Simulation by Manually Specifying Initial Variable Values 16

6 Quantitative Simulation **16**

7 Perform Qualitative, Semi-quantitative, and Quantitative Simulation Using the Same Model **18**

8 Future of JMorven **19**

A The Description of the Physical Systems **19**
A.1 The Single Tank System 19
A.2 The Spring-mass System 21

B JMorven Files **21**
B.1 The JMorven Model File 21
B.2 The Quantity Spaces File 22
B.3 The Initial Values File 23
B.4 The SemiQ Simulation Results File 24

List of Figures

1 JMorven Main Window 5
2 Load Model file 7
3 A Dialog Box Asking for Options 8
4 Total Envisionment for the Single Tank System 8
5 envisionment.txt 9
6 Set Up Values for Exogenous Variables 10
7 Complete Envsionment for the Single Tank System with Steady Input . . . 10
8 Choose An Initial State for Simulation 12
9 Stepwise Qualitative Simulation 13
10 Specify the Step Size for SemiQ Simulation 14
11 Choose the Simulation Mode for SemiQ Simulation 14
12 Choose the Integration Mode for SemiQ Simulation 15
13 Semi-quantitative Simulation Results 15
14 A Zoomed-in Chart Showing the Semi-quantitative Simulation Results . . . 16
15 Manually Specify Initial Values 17
16 Quantitative Simulation for the Spring System 17
17 Quantitative Simulation for the Spring System 18
18 Quantitative Simulation After Three Steps 19
A.1 The Single Tank System 20
A.2 The Spring-mass System 21
B.1 The Model File for the Single Tank System "SingleTank_SimpleQS.txt" . . 22
B.2 The Quantity Spaces File Used for Qualitative Simulation of the Single Tank System (SimpleQS.txt) 23
B.3 The Initial Values File Generated for Qualitative Simulation of the Single Tank Systems (Initial_State.txt) 23
B.4 The Initial Values File Used for Semi-quantitative Simulation of the Spring System (Initial_StateSemiQ_SpringSystem.txt) 24
B.5 The SemiQ Simulation Results File for the Spring Systems (SemiQResult-sXXXXXXXX.csv) 25

List of Tables

1 The *Morven* Model for the Single Tank System 20
2 The Signs Quantity Space 20
3 Function Mappings Under the Signs Quantity Space 21
4 The *Morven* Model for the Spring-Mass System 22

1 Introduction

JMorven is a Java implementation of the *Morven* framework, which was originally proposed by Prof. George M. Coghill [1, 2] and formerly known as the *Mycroft* framework. JMorven was first implemented by Dr. Allan M. Bruce [3, 4], and since 2007, it was maintained and further developed by Dr. Wei Pang. You may find different versions of JMorven, and make sure you are using the latest version, which is **JMorven V1.1a** at the time of writing this user guide.

This user manual focuses on introducing JMorven as a software. To further understand the design principles and algorithm descriptions of JMorven, the reader is directed to references [4] and [5].

2 Installation

2.1 JDK or JRE

Before running JMorven, make sure you have JRE (Java Runtime Enviroment) or JDK (Java Development Kit) installed on your computer. It is noted that JDK contains JRE, so there is no need to install both of them. To check whether your computer have JRE/JDK installed, open a terminal, and type the following command:

java -version

If there is version information shown in the terminal, JRE/JDK is already installed in your computer. Otherwise, go to `www.java.com` and download the latest version of JRE/JDK to your computer. The installation of JRE/JDK is straightfoward.

2.2 Running JMorven

JMorven is distributed as an executable JAR (Java ARchive) file, which means you can run JMorven at any Java enabled platforms. The name of the jar file will be like JMorvenXXX.jar, where XXX is the version information. To run JMorven, open a terminal in Linux, Unix, or MacOS, or a command prompt (cmd.exe) in a Windows PC, "cd" to the folder where the JMorven JAR file is stored, and type the following command:

java -jar JMorvenXXX.jar

You will see a window popping out as shown in Figure 1. This is the main window of JMorven containing all functions.

It is noted that the terminal used to invoke JMorven should always remain open, because in the future any information including error messages from JMorven will be displayed

in this terminal. Consequently, although technically possible, it is not recommended to run the JMoven JAR file by directly double clicking it, as you will not get the terminal to show information from JMorven.

It is also noted that in the terminal you must "cd" to the JMorven program folder first before invoking the JAR file. This is because only by doing so all relevant files generated by JMorven, such as the simulation results file, can be stored in the JMorven program folder.

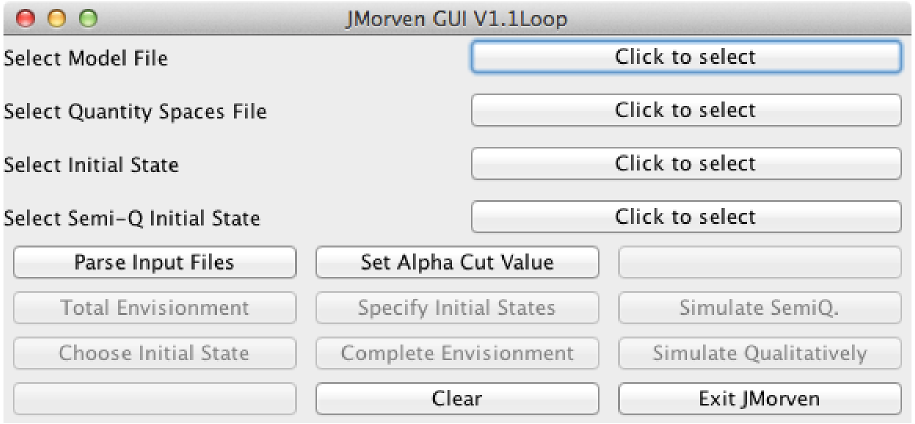

Figure 1: JMorven Main Window

3 Introduction to the JMorven Main Window

All buttons shown in the main window (Figure 1) and their meanings are listed as follows:

- *Select Model File*: select a *Morven* model to be simulated.
- *Select Quantity Spaces File*: select a file defining quantity spaces for variables.
- *Select Initial State*: select a file containing initial values for exogenous variables.
- *Select Semi-Q Initial State*: select a file defining the initial state for semi-quantitative simulation.
- *Parse Input files*: perform the parsing for the model, quantity spaces, and initial state (if available) files.
- *Set Alpha Cut Value*: set the alpha cut value for fuzzy numbers, otherwise a default alpha cut value will be used. It is noted that if we do not perform fuzzy qualitative simulation this function can be ignored.

- *Total Envisionment*: perform the total envisionment (consider all possible qualitative values for all exogenous variables).
- *Specify Initial State*: specify initial values for exogenous variables before performing complete envisionment.
- *Simulate SemiQ*: perform semi-quantitative simulation.
- *Chose Initial State*: chose a particular initial state from the envisionment for qualitative simulation.
- *Complete Envisionment*: perform the complete envisionment (exogenous variables are given initial values)
- *Simulate Qualitatively*: perform the qualitative simulation for a given number of steps.
- *Clear*: clear the memory, and be ready for the next simulation.
- *Exit*: quit JMorven.

4 Qualitative Simulation

In this section we will demonstrate qualitative simulation with JMorven. We use the single tank system as an example to illustrate how to perform the simulation. The single tank system is described in Appendix A.1.

4.1 Load Model File

Click the "Select Model File" button, and a file dialog will be opened, as shown in Figure 2. Go to the "Data/SingleTankQ" subfolder and find a file named "SingleTank_SimpleQS.txt". The meaning of this file is explained by Figure B.1 in Appendix B.1.

4.2 Load Quantity Spaces File

Click the "Select Quantity Spaces File" button and in the popped file dialog select the file named "simpleQS.txt" in the "Data/SingleTankQ" subfolder. The meaning of this file is explained in Figure B.2 of Appendix B.2.

4.3 Parse Input Files

Click the "Parse Input Files" button and parse all input files, including the model, quantity spaces, and initial state (if available) files. If there is any error message shown in the terminal, go back and check the input files. If there is no error, you will see the model and

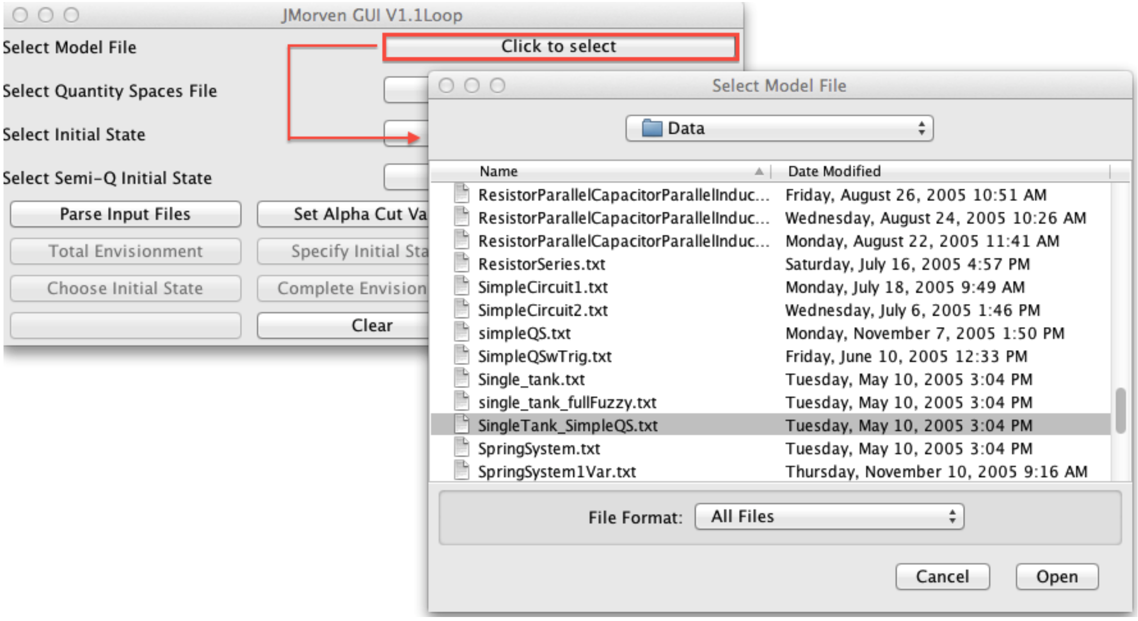

Figure 2: Load Model file

quantity spaces information is displayed in the terminal. If all files are parsed correctly, the "Total Envisionment", "Specify Initial States", and "Simulate SemiQ" buttons will be enabled.

4.4 Total Envisionment

The output of qualitative simulation is called an *envisionment*, which is a directed graph and contains all qualitative states and their legal transitions. In *total envisionment* all possible values of exogenous variables will be considered, and in *complete envisionment* each exogenous variable is given a specific value. Click the button "Total Envisionment", and a dialog box will be shown asking whether to calculate legal transitions between qualitative states in the envisionment, as shown in Figure 3.

If "Yes" is clicked, a second dialog box will be shown asking whether to show the graph. Click "Yes" again and the total envisionment will be displayed, as shown in Figure 4.

In Figure 4 each node represents a qualitative state, and each edge stands for a legal transition from one state to another. Edges are directed from thick to thin to represent the directions of transitions. You can drag and drop all the nodes and arrange them in a nicer way. The green node means that the corresponding state is a steady one. Click each node and the information about this state will be displayed in the right part of the window. The information includes qualitative values assigned to all variables, the UID (Unique ID)

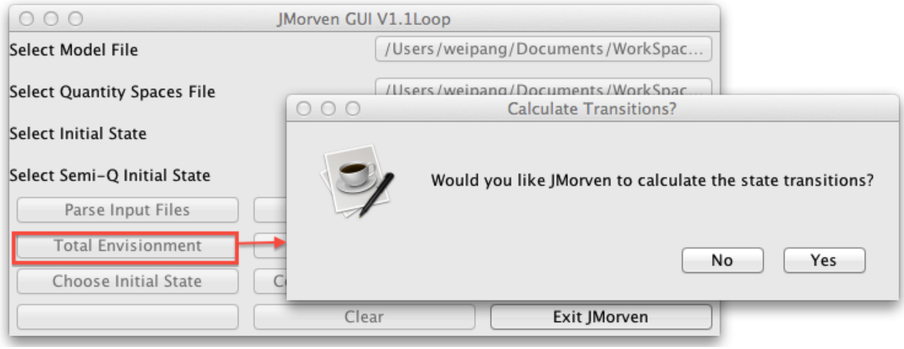

Figure 3: A Dialog Box Asking for Options

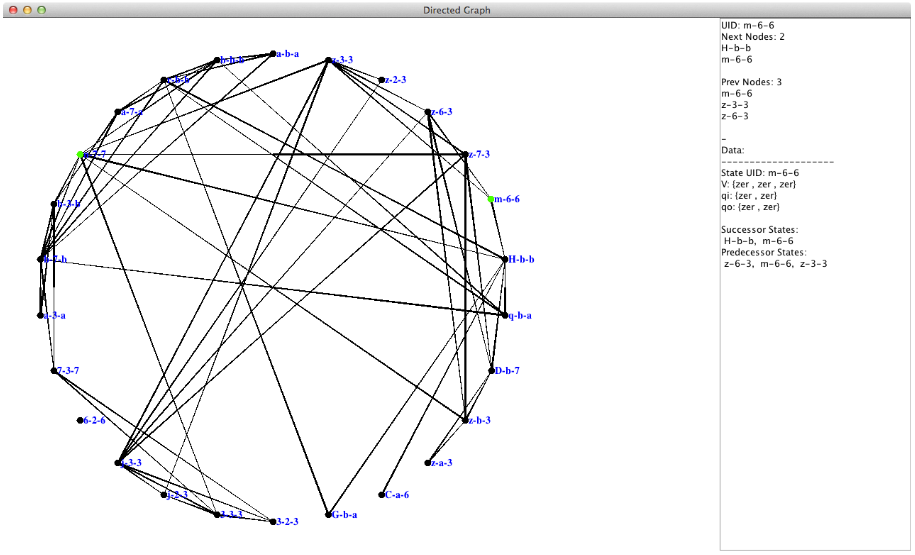

Figure 4: Total Envisionment for the Single Tank System

of this qualitative state calculated from a hash function which takes qualitative values as arguments, and the UIDs of the successor and predecessor states in the envisionment. For instance, in the right part of Figure 4 the state with the UID "m-6-6" is currently shown, and the qualitative values of all variables are also given in the form of vectors. In this state, V={pos, zer, zer} means that the magnitude of Variable V is *positive*, the first derivative of V is *zero*, and the second derivative of V is also *zero*. Qualitative values *pos*, *zer*, and *neg* are defined in the quantity spaces file SimpleQS.txt, as mentioned in Section 4.2. The assignment of variable V indicates that in State"m-6-6" the volume of the

tank is positive and remains unchanged. From Figure 4 we can also see that State "m-6-6" has two successor states and three predecessor states.

After qualitative simulation of the single tank system, a file called *envisionment.txt* is generated and stored in the JMorven program folder. In this file all information about the envisioment are stored for further use. A snapshot of the file is shown in Figure 5.

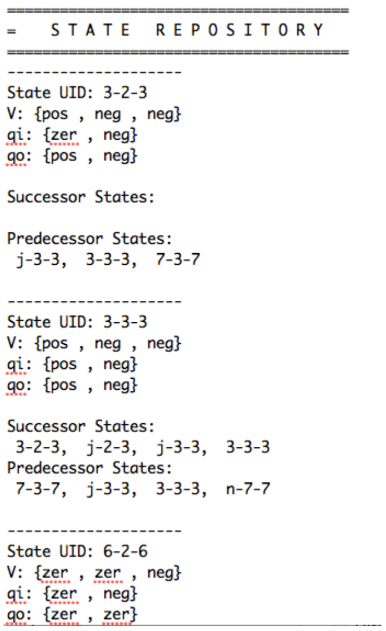

```
====================================
=   S T A T E   R E P O S I T O R Y
====================================
--------------------
State UID: 3-2-3
V: {pos , neg , neg}
qi: {zer , neg}
qo: {pos , neg}

Successor States:

Predecessor States:
 j-3-3,  3-3-3,  7-3-7

--------------------
State UID: 3-3-3
V: {pos , neg , neg}
qi: {pos , neg}
qo: {pos , neg}

Successor States:
 3-2-3,  j-2-3,  j-3-3,  3-3-3
Predecessor States:
 7-3-7,  j-3-3,  3-3-3,  n-7-7

--------------------
State UID: 6-2-6
V: {zer , zer , neg}
qi: {zer , neg}
qo: {zer , zer}
```

Figure 5: envisionment.txt

4.5 Complete Envisionment

As mentioned before, in *complete envisionment* we give each exogenous variable a specific value and perform the simulation. In this section we will demonstrate the complete envisionment when the input flow q_i of the single tank is set to positive and steady.

First, click the "Clear" button in the JMorven main window, and this will clear the previous simulation results. Second, click the "Parse Input Files" button and reload the model and quantity spaces files. Third, click the "Specify Initial States" button, and a window for setting up the values of exogenous variables will show up. Set the value of the only exogenous variable q_i to be <pos, zer>, as shown in Figure 6. Remember to tick the "fixed" box, which means we want the value of q_i to be fixed throughout the simulation.

After setting up the values for exogenous variables, click the "Complete Envisionment" button, and the simulation results are shown in Figure 7. From this figure we see that

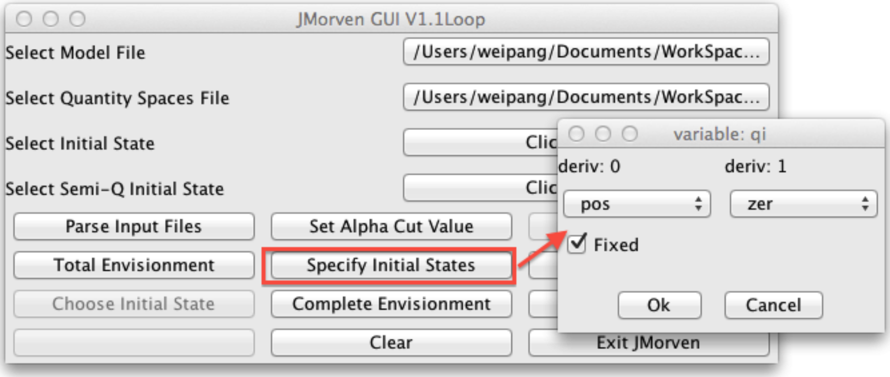

Figure 6: Set Up Values for Exogenous Variables

there are only four qualitative states generated in the envisionment graph. Again you can drag and drop the nodes in the graph, and find the generated "envisionment.txt" for this simulation.

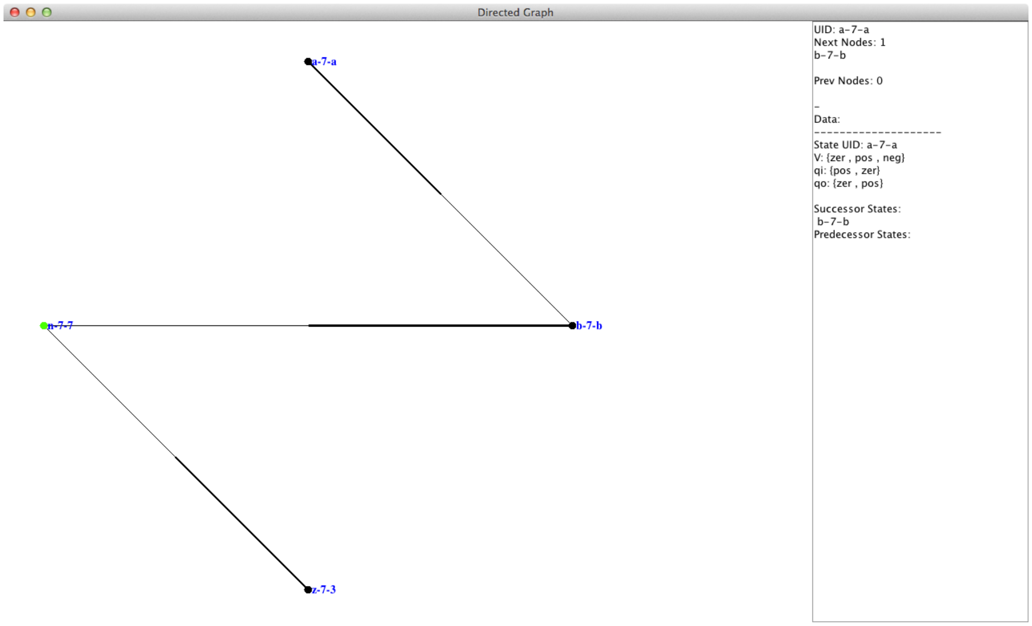

Figure 7: Complete Envsionment for the Single Tank System with Steady Input

It is noted that after specifying the values for exogenous variables, a file named "Ini-

tial_State.txt" will be generated and stored in the JMorven program folder to record these values. A snapshot and description of this file for the simulation of the Singe Tank System is shown in Figure B.3 of Appendix B.3. In the future simulation you can load this file by clicking the "Select Initial State" button instead of manually specifying values to perform complete envisionment.

4.6 Stepwise Qualitative Simulation

We can also perform qualitative simulation step by step, which enables us to study a particular part of the envisionment. To better demonstrate this, we use the Spring-mass System (described in Appendix A.2) as an example because it has more complicated behaviours than the Single Tank.

4.6.1 Load Model File and Quantity Spaces File

The model and quantity spaces files are in the "data/SpringQ" subfolder and called "SpringSystemSimpleQS.txt" and "simpleQS.txt", respectively. Load these two files as we did in Sections 4.1 and 4.2.

4.6.2 Complete Envisionment

Similar to Figure 6, click "Specify Initial States" and set the value of F to be <zer, zer>, and tick the "Fixed" box.

Click the "Complete Envisionment" button, and you will get the envisionment graph as before.

4.6.3 Choose Initial State

Click "Choose Initial State", and you will be asked to choose an initial state for the simulation, as shown in Figure 8.

Choose State 0 and click "Ok". The window will disappear and we will come back to the JMorven Main Window.

4.6.4 Perform the Simulation

Click the "Simulate Qualitatively" button in the JMorven main window, and in the next pop-up window set the number of steps to be 7.

The simulation will start, and after the simulation is finished the results are shown in Figure 9. In this figure we can see that the simulation starts from the state represented by a red point and after seven steps the system reaches State "a-6-q-7".

After the simulation a file named "simulation.txt"" is generated and stored in the JMorven program folder. The format of this file is similar to "envisionment.txt" shown in Figure 5, except that the start state is recorded in this file.

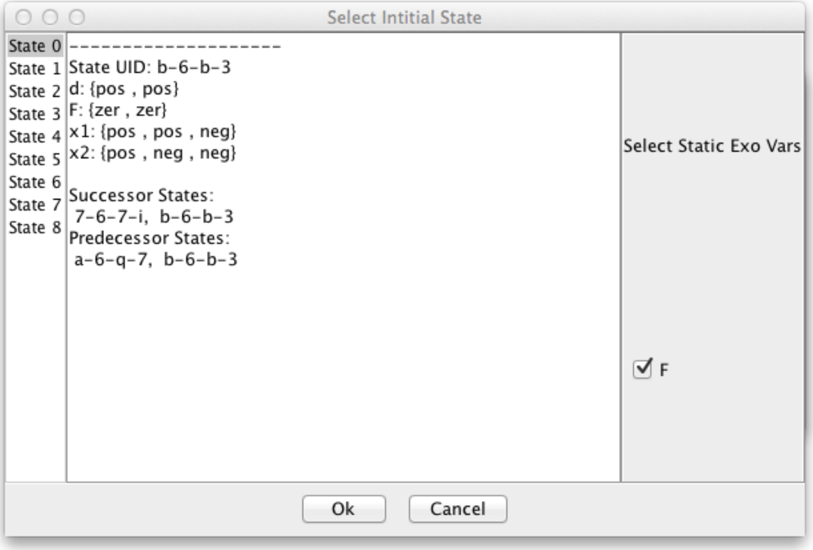

Figure 8: Choose An Initial State for Simulation

5 Semi-quantitative Simulation

We use the same Spring-mass System as in Section 4.6 to demonstrate semi-quantitative (SemiQ) simulation.

5.1 Load Model File and Quantity Spaces File

The model and quantity spaces files are in the "data/SpringSemiQ" subfolder and called "SpringSystem.txt" and "qspacesNew.txt", respectively. Load this two files as we did in Sections 4.1 and 4.2.

5.2 Perform Simulation Using a Predefined Initial Values File

In order to perform semi-quantitative simulation, we also need to specify the initial values for some of the variables. This can be done in two ways: (1) load a predefined file containing initial SemiQ values for variables, or (2) manually specify initial values. In this section, we will describe the first way, and in Section 5.3, we will introduce the second way.

In the same "data/SpringSemiQ" subfolder find a file named *Initial_StateSemiQ_SpringSystem.txt*. A snapshot and detailed explanation of this file is given in Figure B.4 of Appendix B.3.

It is noted that this file can be either coded by hand or automatically generated by JMorven, which will be described in Section 5.3. Click the "Select SemiQ Initial State" button and load this file.

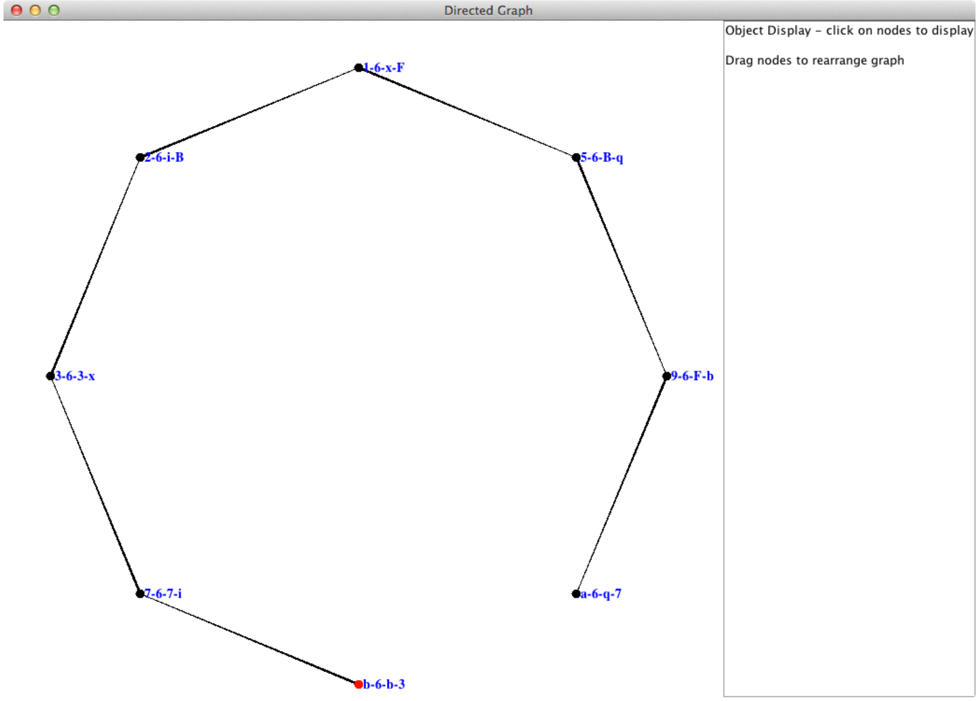

Figure 9: Stepwise Qualitative Simulation

Click the “Parse Input Files” button, and if there is no error reported from the terminal, the “Simulate SemiQ” button will be enabled. Click this button, and you will be asked to specify the step size for the simulation, as shown in Figure 10. Set the step size to be 0.01, and you will be asked to specify the length of time to simulate. Set the time to be 10, and you will be asked to select the simulation mode, as shown in Figure 11. We can see that there are a number of different simulation modes available. For more details about these simulation modes, please refer to reference [5]. Select ”Monte Carlo Point Simulation”, and the next window will ask you to choose the integration method, as shown in Figure 12. More details about these integration methods are available in [5]. Select the ”Taylor Method”. Because we choose the ”Monte Carlo Point Simulation”, we should also provide the number of Monte Carlo iterations in the next window. The bigger this number, the more precise results we will get, and the longer simulation time it will take. For the purpose of demonstration, we give a small number 10 to make the simulation quicker.

After specifying all the above parameters, the semi-quantitative simulation will start. A progress bar may appear indicating the current progress of the simulation. After the simulation has been finished, you can choose which variables to be plotted in the chart. Select “x1” and “x2”, and the final simulation results are shown in Figure 13. In the chart

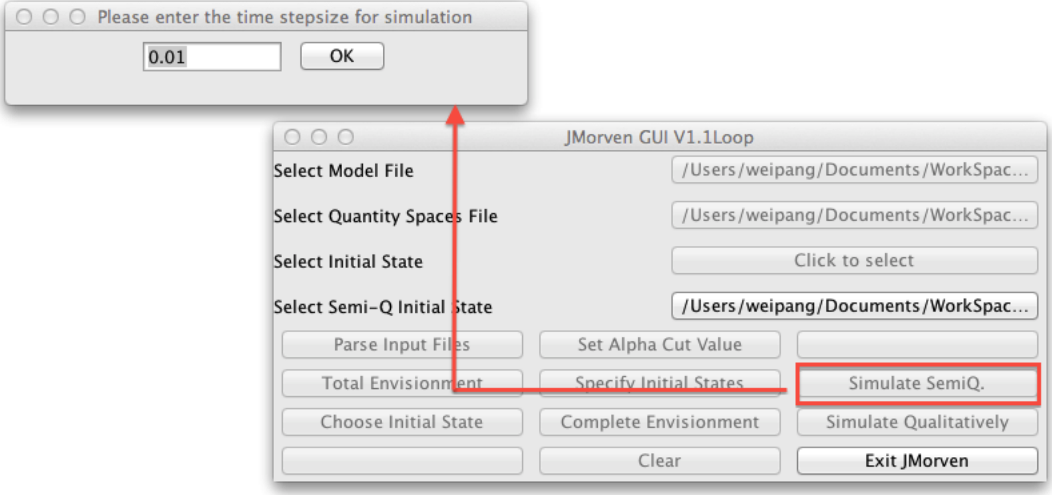

Figure 10: Specify the Step Size for SemiQ Simulation

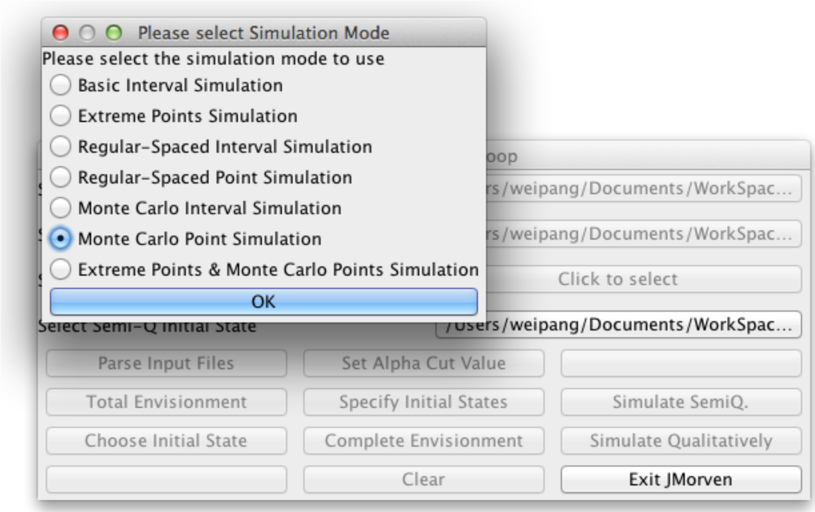

Figure 11: Choose the Simulation Mode for SemiQ Simulation

shown in Figure 13, you can either get more detailed simulation information by hovering the cursor over a specific point of the curve, or zoom in a particular area by using the mouse to select this area. A zoomed-in version of the chart is shown in Figure 14. In

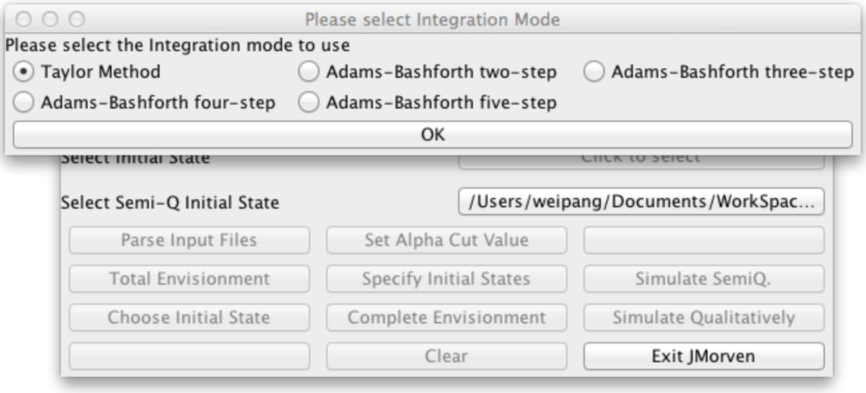

Figure 12: Choose the Integration Mode for SemiQ Simulation

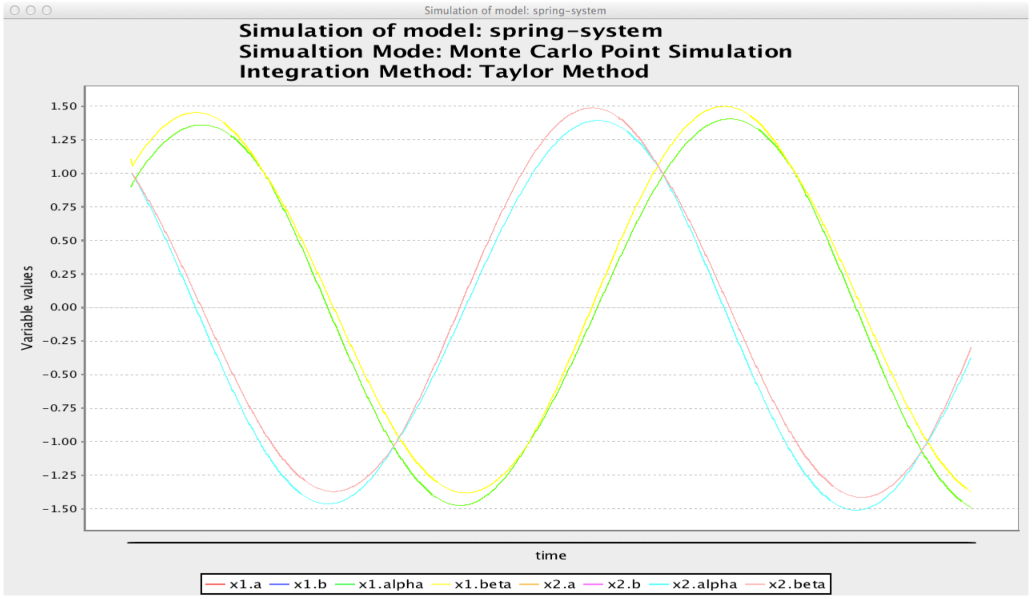

Figure 13: Semi-quantitative Simulation Results

addition, the simulation results are store in a file named "SemiQResultsXXXXXX.csv", where "XXXXXX" is the time stamp. An example of the SemiQ results file is given in Figure B.5 of Appendix B.4.

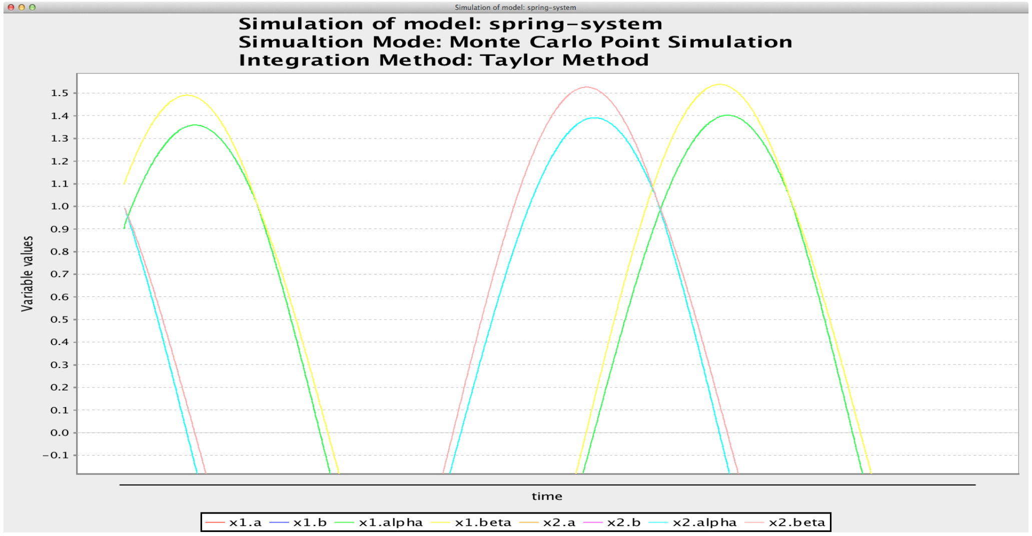

Figure 14: A Zoomed-in Chart Showing the Semi-quantitative Simulation Results

5.3 Perform Simulation by Manually Specifying Initial Variable Values

When the file for initial variable values is not specified and after the "Simulate SemiQ." button is clicked, several windows will be displayed and you can specify initial values for some of the variables through these windows. One of such windows is shown in Figure 15. In this window we specify that the magnitude of F is <0, 0, 0, 0>. It is noted that F is in the form of fuzzy four tuples <a, b, alpha, beta>. In this window we also see that the first derivative of F remain unspecified. After the specification of initial variable values, the rest of the operations remain the same as in Section 5.2.

It is noted that after specifying initial values, a file called "Initial_StateSemiQ.txt" will be generated and stored in the JMorven program folder. This newly generated file can be used for future simulations as a predefined file, as described in Section 5.2.

6 Quantitative Simulation

Quantitative Simulation is considered as a special case of semi-quantitative simulation in JMorven, because we can perform quantitative simulation by using the same semi-quantitative simulation algorithm and setting the initial values to be zero-width intervals. It is noted that in the current version of JMorven certain simulation modes may not be available for quantitative simulation, including "Extreme Point Simulation", "Regular Spaced Interval Simulation", and "Regular Spaced Point Simulation". It is recommend to use the "Basic Interval Simulation" mode for quantitative simulation.

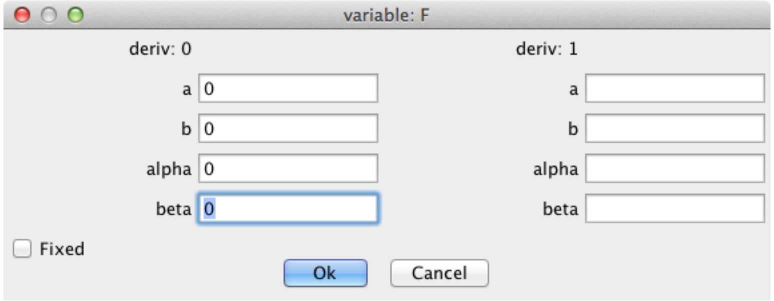

Figure 15: Manually Specify Initial Values

We perform the quantitative simulation for the same Spring-mass System as in Section 5.2. We use the same model and quantity spaces files as in Section 5.2, and in the subfolder "data/SpringSemiQ" select the initial SemiQ values file to be "Initial_StateQuantitative_SpringSystem.txt", in which all variables are set to be zero-width intervals. We choose the "Basic Interval Simulation" mode. The rest configurations of the simulation are the same as those in Section 5.2. Finally Figure 16 shows the quantitative simulation results for the Spring System.

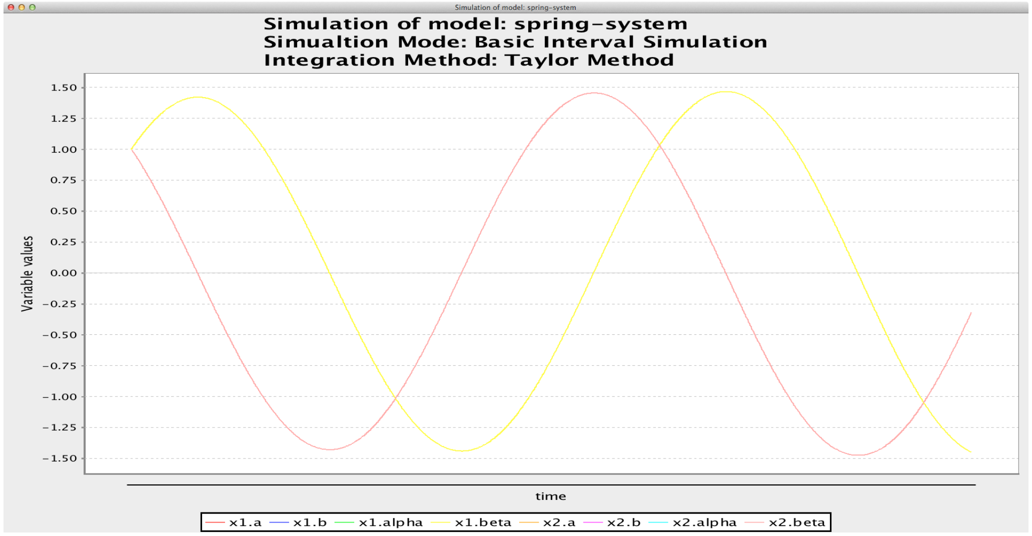

Figure 16: Quantitative Simulation for the Spring System

7 Perform Qualitative, Semi-quantitative, and Quantitative Simulation Using the Same Model

An important feature of JMorven is to perform all three kinds of simulation using the same model. In Section 5 and Section 6 we perform semi-quantitative and quantitative simulation using the same Spring-mass Model described by the files “SpringSystem.txt” and “qspacesNew.txt” in the data/SpringSemiQ subfolder.

In this section, we will perform qualitative simulation using the same model and quantity spaces files. Figure 17 shows the complete envisionment when F is set to <zer, zer>, and Figure 18 shows the qualitative simulation starting from State “5-p-t1-Z1” after three steps. It is noted that in Figure 18 the nodes have been rearranged in a nicer way.

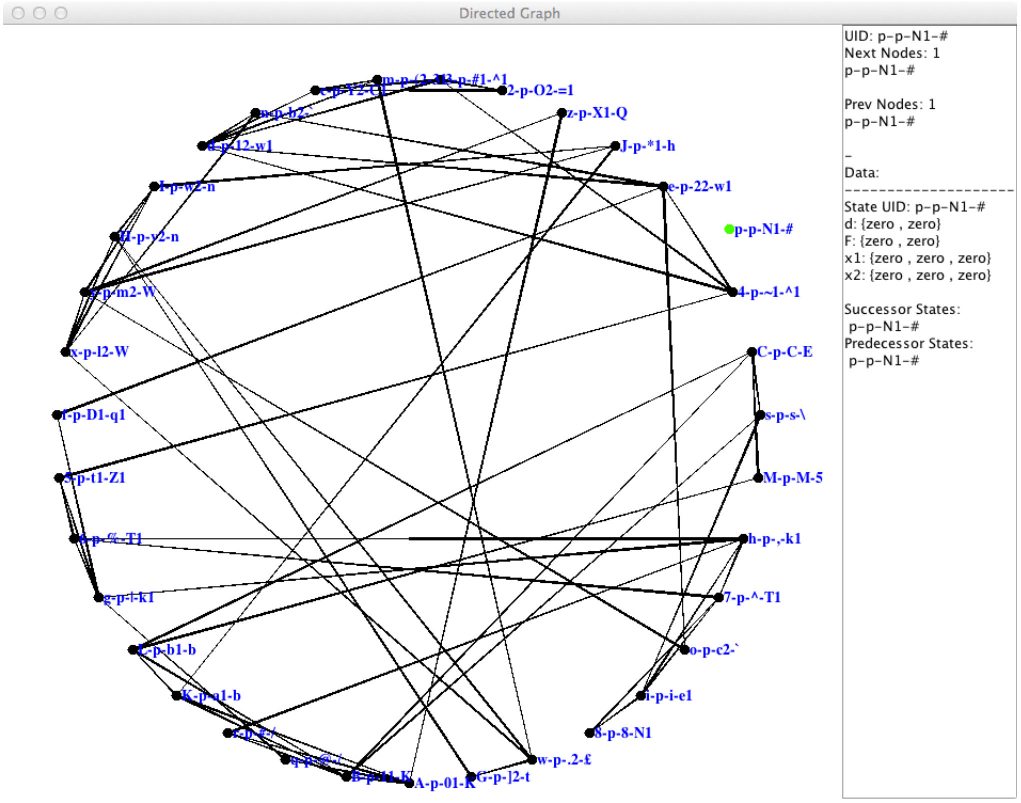

Figure 17: Quantitative Simulation for the Spring System

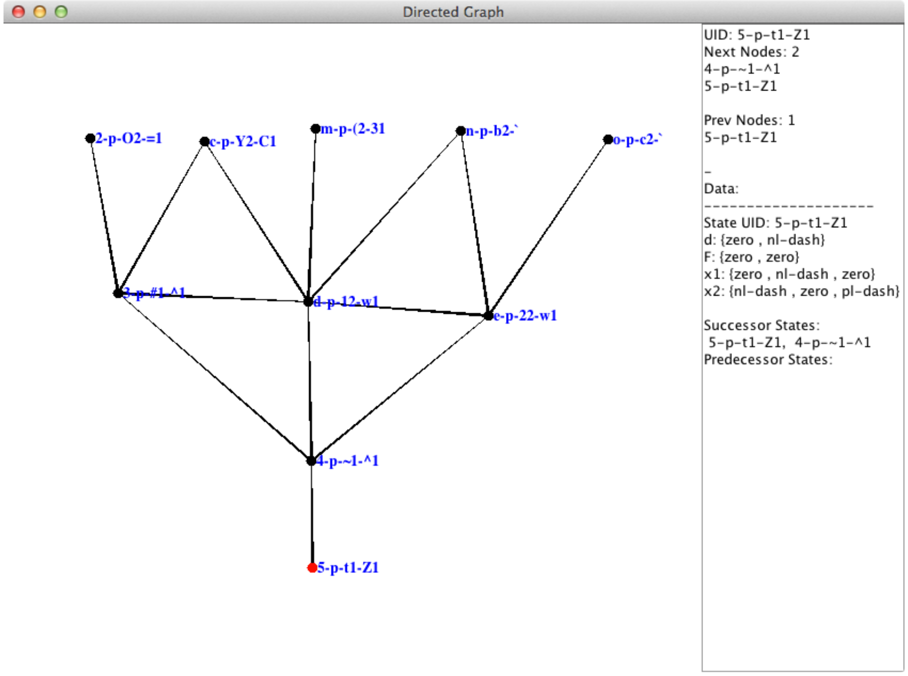

Figure 18: Quantitative Simulation After Three Steps

8 Future of JMorven

Currently many components for qualitative and semi-quantitative simulation have been parallelised by using the Java multi-threading technology. This enables JMorven to make better use of a computer with multiple processors. In the future we will further make JMoren adapt to cluster computing and cloud computing environments.

A The Description of the Physical Systems

A.1 The Single Tank System

The single tank system is shown in Figure A.1. The numerical model for a linear version of this system is given by Equations (1) and (2).

$$q_o = k * V, \tag{1}$$

$$dV/dt = q_i - q_o. \tag{2}$$

In the above equations V is the volume of the liquid in the tank, q_i is the inflow, q_o is

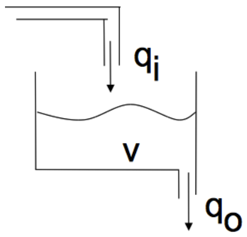

Figure A.1: The Single Tank System

Table 1: The *Morven* Model for the Single Tank System

Differential Plane 0	
C1: Function (dt 0 q_o, dt 0 V)	$(q_o = k * V)$
C2: sub (dt 1 V, dt 0 q_i, dt 0 q_o)	$(V' = q_i - q_o)$
Differential Plane 1	
C3: Function (dt 1 q_o, dt1 V)	$(q_o' = k * V')$
C4: sub (dt 2 V, dt1 q_i, dt1 q_o)	$(V'' = q_i' - q_o')$

the outflow, and k is a positive constant coefficient determined by the cross sectional area of the tank and the density of the liquid.

The corresponding *Morven* model is shown in Table 1. This model is composed of four constraints, *C1* to *C4*. The meaning of these constraints has been explained in [4, 5] , and the corresponding quantitative relation for each constraint is shown on the right hand side in the brackets. For variable V, the magnitude, the first and second derivatives are used; for variable q_o and q_i, only the magnitude and the first derivative are used.

If all the qualitative variables (including their magnitudes and derivatives) use the signs quantity space, which is shown in Table 2, the mappings of the *Function* in constraint *C1* and *C3* are given in Table 3, in which "1" stands for the existence of a mapping between variables A and B.

Table 2: The Signs Quantity Space

Quantity	Range	
negative(-)	$(-\infty,$	$0)$
zero(0)	0	
positive(+)	$(0,$	$\infty)$

Table 3: Function Mappings Under the Signs Quantity Space

Function(A,B)	negative	zero	positive
negative	1	0	0
zero	0	1	0
positive	0	0	1

A.2 The Spring-mass System

The Spring-mass System, or the Spring System for short, is shown in Figure A.2. This system can be modelled by the following equation:

$$x'' = F - kx \tag{3}$$

In the above F is the external force which is assumed to be constant, k is a constant parameter related to the mass and the spring, and x is the displacement of the mass with respect to the equilibrium position. The *Morven* model for the Spring-mass System is

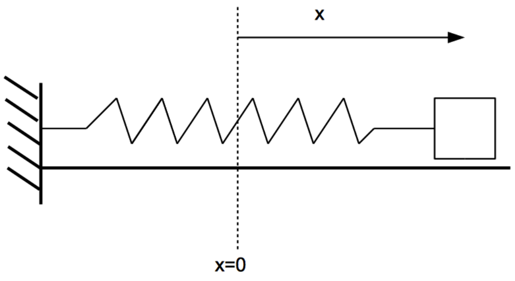

Figure A.2: The Spring-mass System

given in Table 4.

B JMorven Files

B.1 The JMorven Model File

A JMorven model file defines a *Morven* model, including variables, quantity spaces associated with these variables, and all qualitative constraints distributed across different differential planes. Figure B.1 gives a detailed explanation of the JMorven model file for the single tank system.

Table 4: The *Morven* Model for the Spring-Mass System

Differential Plane 0
C1: func (dt 0 d, dt 0 x1)
C2: func (dt 1 x1, dt 0 x2)
C3: sub (dt 1 x2, dt 0 F, dt 0 d)
Differential Plane 1
C4: func (dt 1 d, dt 1 x1)
C5: func (dt 2 x1, dt 1 x2)
C6: sub (dt 2 x2, dt 1 F, dt 1 d)

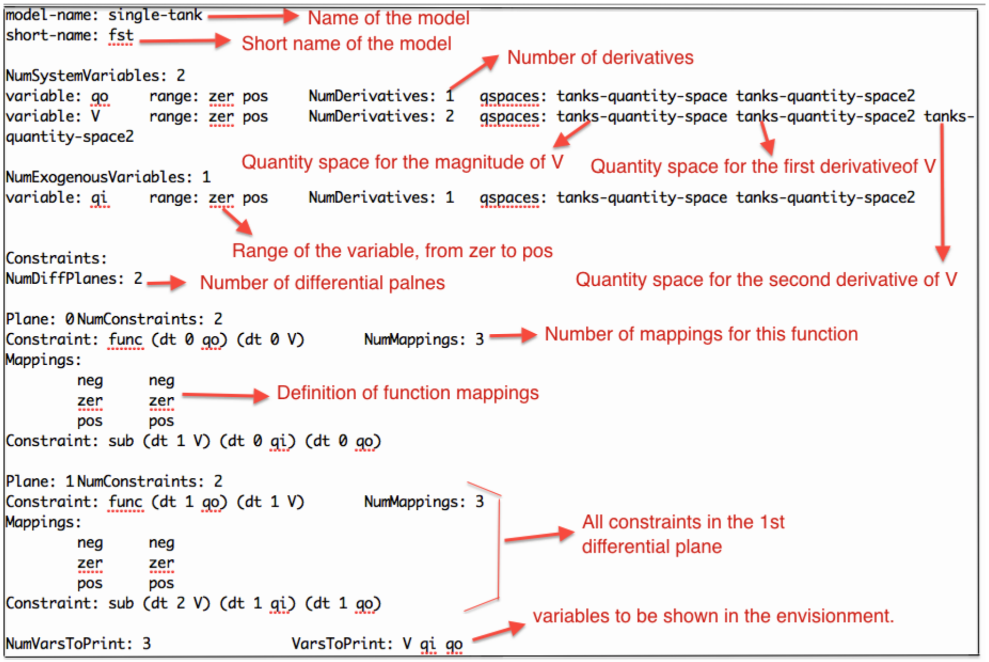

Figure B.1: The Model File for the Single Tank System "SingleTank_SimpleQS.txt"

B.2 The Quantity Spaces File

The quantity spaces file defines all the quantity spaces used by qualitative variables. Figure B.2 explains the meaning of the quantity spaces file used for simulating the single tank system. In this file there are four quantity spaces defined. Each quantity in the quantity space is defined by a fuzzy number in the form of four tuple <*a*, *b*, *alpha*, *beta*>, as in [6]. If *alpha* and *beta* are zero, the quantity becomes a non-fuzzy interval, which is the case for all simulations presented in this manual.

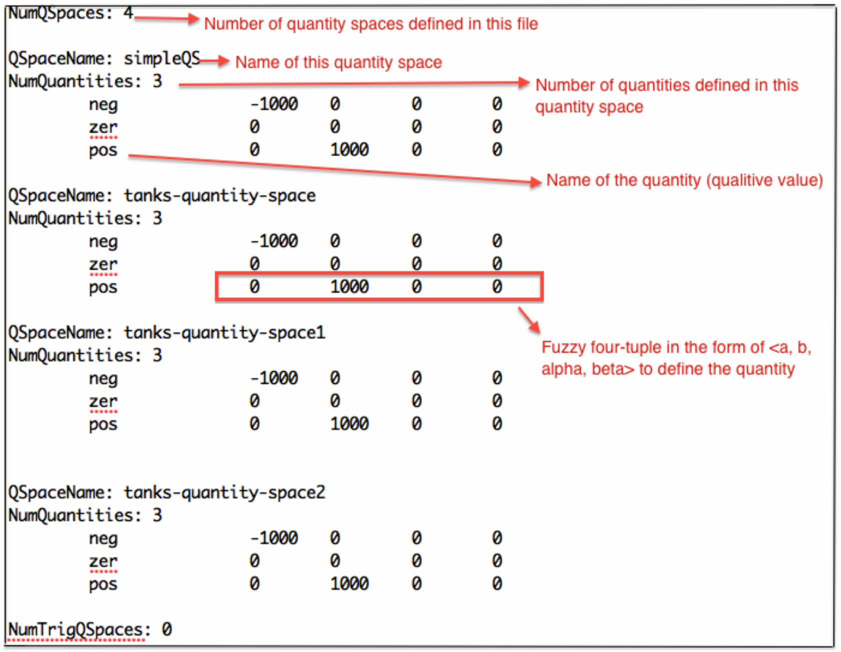

Figure B.2: The Quantity Spaces File Used for Qualitative Simulation of the Single Tank System (SimpleQS.txt)

B.3 The Initial Values File

For qualitative simulation, the initial values file is generated after the user clicks the "Specify Initial States" button and specifies the initial values for exogenous variables. If the user specifies q_i to be $<pos, zer>$ (positive and steady), the initial values file and its meaning is shown in Figure B.3.

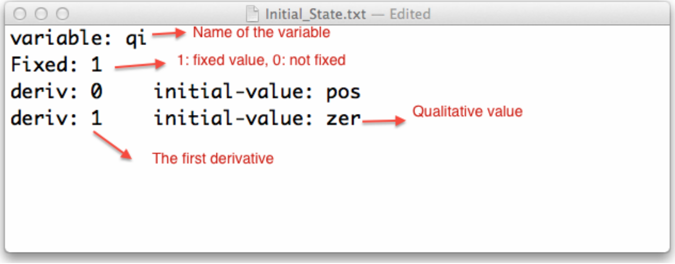

Figure B.3: The Initial Values File Generated for Qualitative Simulation of the Single Tank Systems (Initial_State.txt)

For semi-quantitative and quantitative simulation, the initial values file is generated after the user does not click the “Select SemiQ Initial State” button, but clicks the “Simulate SemiQ” button and manually specifies the initial values for simulation.

A snapshot of the initial values file for semi-quantitative simulation of the Spring-mass System is shown in Figure B.4.

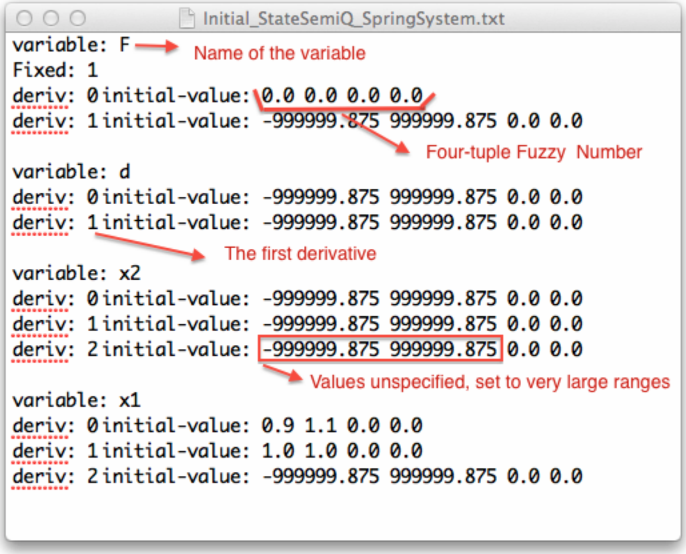

Figure B.4: The Initial Values File Used for Semi-quantitative Simulation of the Spring System (Initial_StateSemiQ_SpringSystem.txt)

B.4 The SemiQ Simulation Results File

After each semi-quantitative simulation, a file named “SemiQResultsXXXXXXXX.csv” is generated to record all the simulation results and stored in the JMorven program folder, where “XXXXXXXX”” is a time stamp to distinguish this file from others. A snapshot of the SemiQ simulation results file for the Spring-mass System is given in Figure B.5.

References

[1] Coghill, G.M.: Mycroft: A Framework for Constraint based Fuzzy Qualitative Reasoning. PhD thesis, Heriot-Watt University (September 1996)

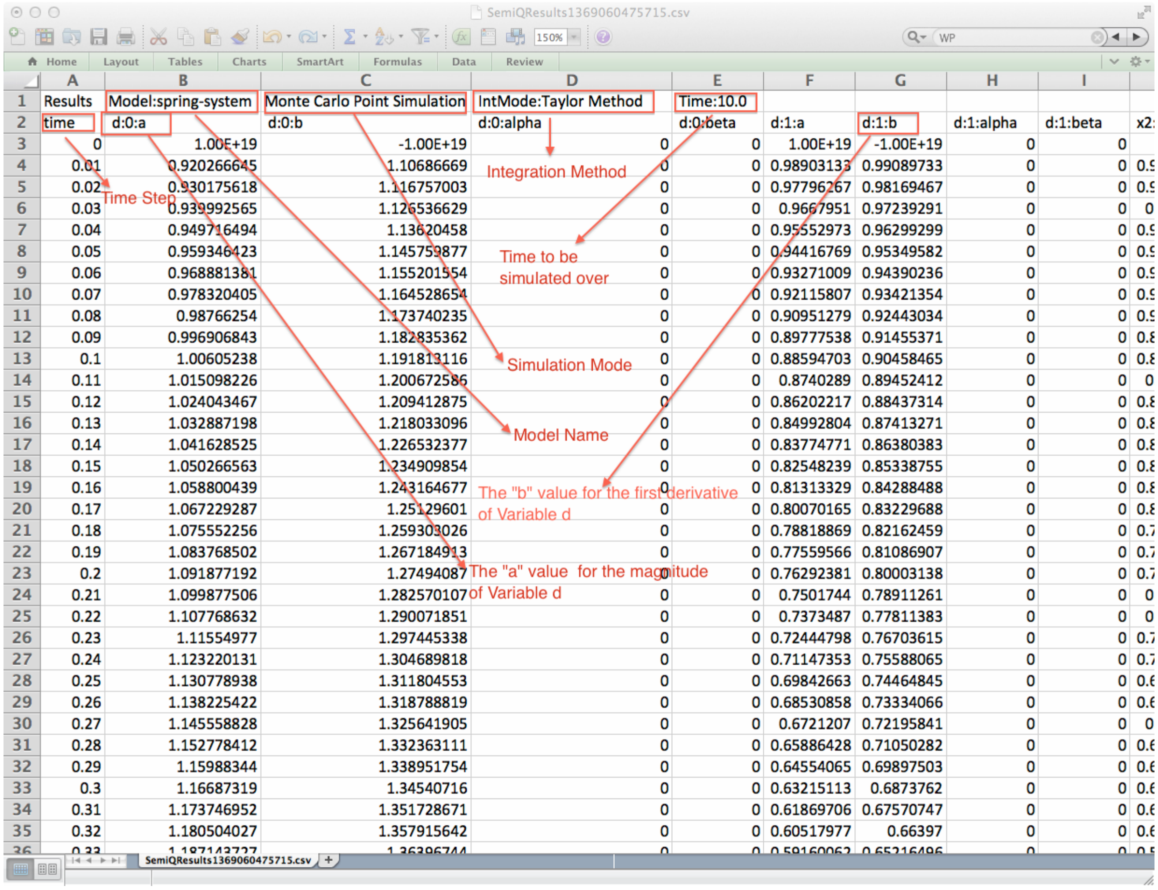

	A	B	C	D	E	F	G	H	I	
1	Results	Model:spring-system	Monte Carlo Point Simulation	IntMode:Taylor Method	Time:10.0					
2	time	d:0:a	d:0:b	d:0:alpha	d:0:beta	d:1:a	d:1:b	d:1:alpha	d:1:beta	x2:
3	0	1.00E+19	-1.00E+19	0	0	1.00E+19	-1.00E+19	0	0	
4	0.01	0.920266645	1.10686669	0	0	0.98903133	0.99089733	0	0	0.9
5	0.02	0.930175618	1.116757003	0	0	0.97796267	0.98169467	0	0	0.9
6	0.03	0.939992565	1.126536629	0	0	0.9667951	0.97239291	0	0	0
7	0.04	0.949716494	1.13620458	0	0	0.95552973	0.96299299	0	0	0.9
8	0.05	0.959346423	1.145759877	0	0	0.94416769	0.95349582	0	0	0.9
9	0.06	0.968881381	1.155201554	0	0	0.93271009	0.94390236	0	0	0.9
10	0.07	0.978320405	1.164528654	0	0	0.92115807	0.93421354	0	0	0.9
11	0.08	0.98766254	1.173740235	0	0	0.90951279	0.92443034	0	0	0.9
12	0.09	0.996906843	1.182835362	0	0	0.89777538	0.91455371	0	0	0.8
13	0.1	1.00605238	1.191813116	0	0	0.88594703	0.90458465	0	0	0.8
14	0.11	1.015098226	1.200672586	0	0	0.8740289	0.89452412	0	0	0
15	0.12	1.024043467	1.209412875	0	0	0.86202217	0.88437314	0	0	0.8
16	0.13	1.032887198	1.218033096	0	0	0.84992804	0.87413271	0	0	0.8
17	0.14	1.041628525	1.226532377	0	0	0.83774771	0.86380383	0	0	0.8
18	0.15	1.050266563	1.234909854	0	0	0.82548239	0.85338755	0	0	0.8
19	0.16	1.058800439	1.243164677	0	0	0.81313329	0.84288488	0	0	0.8
20	0.17	1.067229287	1.25129601	0	0	0.80070165	0.83229688	0	0	0.8
21	0.18	1.075552256	1.259303026	0	0	0.78818869	0.82162459	0	0	0.7
22	0.19	1.083768502	1.267184913	0	0	0.77559566	0.81086907	0	0	0.7
23	0.2	1.091877192	1.27494087	0	0	0.76292381	0.80003138	0	0	0.7
24	0.21	1.099877506	1.282570107	0	0	0.7501744	0.78911261	0	0	0
25	0.22	1.107768632	1.290071851	0	0	0.7373487	0.77811383	0	0	0
26	0.23	1.11554977	1.297445338	0	0	0.72444798	0.76703615	0	0	0.7
27	0.24	1.123220131	1.304689818	0	0	0.71147353	0.75588065	0	0	0.7
28	0.25	1.130778938	1.311804553	0	0	0.69842663	0.74464845	0	0	0.6
29	0.26	1.138225422	1.318788819	0	0	0.68530858	0.73334066	0	0	0.6
30	0.27	1.145558828	1.325641905	0	0	0.6721207	0.72195841	0	0	0
31	0.28	1.152778412	1.332363111	0	0	0.65886428	0.71050282	0	0	0.6
32	0.29	1.15988344	1.338951754	0	0	0.64554065	0.69897503	0	0	0.6
33	0.3	1.16687319	1.34540716	0	0	0.63215113	0.6873762	0	0	0.6
34	0.31	1.173746952	1.351728671	0	0	0.61869706	0.67570747	0	0	0.6
35	0.32	1.180504027	1.357915642	0	0	0.60517977	0.66397	0	0	0.6
36	0.33	1.187143727	1.36396744	0	0	0.59160062	0.65216496	0	0	0.5

Figure B.5: The SemiQ Simulation Results File for the Spring Systems (SemiQResultsXXXXXXXXX.csv)

[2] Coghill, G.M., Chantler, M.J.: Mycroft: a framework for qualitative reasoning. In: Second International Conference on Intelligent Systems Engineering, Hamburg-Harburg, Germany (September 1994) 43 – 48

[3] Bruce, A.M., Coghill, G.M.: Parallel fuzzy qualitative reasoning. In: Proceedings of the 19th International Workshop on Qualitative Reasoning, Graz, Austria (2005) 110–116

[4] Bruce, A.M.: JMorven: A Framework for parallel non-constructive qualitative reasoning and fuzzy interval simulation. PhD thesis, Department of Computing Science, Univeristy of Aberdeen (October 2007)

[5] Pang, W., Coghill, G.M.: Non-constructive interval simulation of dynamic systems. Technique Report ABDN-CS-12-02, Department of Computing Science, University of Aberdeen (2012)

[6] Shen, Q., Leitch, R.: Fuzzy qualitative simulation. IEEE Transactions on Systems, Man, and Cybernetics **23**(4) (1993) 1038–1061
